# Differential regulation of *ZFAS1* splice variants by endoplasmic reticulum stress in hepatocyte cell lines

**DOI:** 10.1002/2211-5463.70185

**Published:** 2026-02-06

**Authors:** Sébastien Soubeyrand, Paulina Lau, Ruth McPherson

**Affiliations:** ^1^ Atherogenomics Laboratory University of Ottawa Heart Institute Canada; ^2^ Division of Cardiology, Ruddy Canadian Cardiovascular Genetics Centre University of Ottawa Heart Institute Canada

**Keywords:** ER stress, hepatocytes, HepG2, *TRIBAL*, unfolded protein response, *ZFAS1*

## Abstract

The suppression of the long noncoding RNA (lncRNA) *TRIBAL* in hepatocytes was recently shown to affect the expression of pivotal regulators and hundreds of poorly understood or uncharacterized transcripts. The most upregulated transcript corresponded to a predicted splice variant of the lncRNA *ZFAS1*. Here, we characterize and investigate the role and regulation of *ZFAS1* splice variants in liver cell models. New *ZFAS1* splice variants were identified, all of which were enriched in the cytoplasm of HepG2 cells. *TRIBAL* suppression strongly upregulated a low‐abundance *ZFAS1* variant in hepatocytes but not in hepatoma models. However, preventing the upregulation of the *ZFAS1* splice variant did not mitigate the impact of *TRIBAL* suppression in hepatocytes. *ZFAS1* variants were rapidly but differentially increased in response to thapsigargin, which causes endoplasmic reticulum (ER) stress and activates the unfolded protein response (UPR). Inhibition of PERK, a central sensor of the UPR, had contrasting impacts on ZFAS1 variants in response to thapsigargin. Moreover, whereas the upregulation of the main *ZFAS1* form was reduced by the suppression of the UPR mediators ATF4 and NFE2L2 (also known as NRF2), the other variants were not. Lastly, *ZFAS1* suppression decreased cell viability both at baseline and in response to acute thapsigargin treatment. This work identifies novel *ZFAS1* variants and uncovers a link between ER stress and *ZFAS1* through the UPR.

## Graphical abstract

*ZFAS1* is a lncRNA promoting cell proliferation and migration, exhibiting high expression in various cancers. It is conserved, widely expressed, and produces multiple splice variants with unclear roles. We identified several splice variants in hepatocyte models, and found that inhibiting or suppressing regulators of the unfolded protein response (PERK, ATF4, NF2EL2) affected them differently under stress, pointing to unique functions.

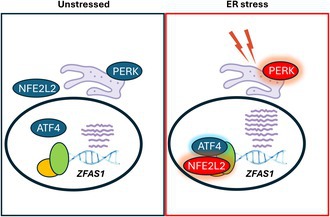

**Abbreviations**
ASO: Antisense oligonucleotide
ATF4: Activating Transcription Factor 4
ATF6: Activating Transcription Factor 6
BiP: binding immunoglobulin protein
CHOP: C/EBP homologous protein
ER: endoplasmic reticulum
FCCP: Carbonyl cyanide 4‐(trifluoromethoxy)phenylhydrazone
HNF4A: Hepatocyte Nuclear Factor 4 Alpha
IRE1: Inositol‐Requiring Enzyme 1.
LG‐DMEM: low glucose Dulbecco's Modified Eagle Medium
LncRNA: long noncoding RNA
MLXIPL: MLX Interacting Protein Like
NFE2L2: NFE2‐Like BZIP Transcription Factor 2
PBS: Phosphate‐buffered saline
PERK: PRKR‐Like Endoplasmic Reticulum Kinase
PMA: Phorbol 12‐myristate 13‐acetate
PPIA: Peptidylprolyl Isomerase A
qRT‐PCR: quantitative real‐time PCR
siRNA: silencing RNA
snoRNA: Small Nucleolar RNA
snRNA: Small Nuclear RNA
TBS: Tris‐buffered saline
Tg: Thapsigargin
TRIBAL: TRIB1 Associated LncRNA
U1: RNU1‐1
UPR: Unfolded protein response
ZFAS1: ZNFX1 Antisense RNA 1

Long noncoding RNAs (lncRNAs) are defined by their length exceeding 200 bp and the absence of protein‐coding potential. Dismissed initially as transcriptional noise, in part due to their lower abundance, the vast majority of lncRNAs remain uncharacterized [1]. The number of lncRNA genes is likely to exceed that of protein‐coding genes, and they are increasingly recognized as contributing to a wide range of physiological and pathological processes [2, 3, 4]. Relative to mRNA, less efficient splicing results in increased lncRNA diversity and the production of functionally distinct and sometimes antagonistic transcripts [4, 5].

Zinc finger antisense 1 (*ZFAS1*) is a conserved lncRNA gene that abuts, in a head‐to‐head configuration, the protein‐coding gene Zinc finger NFX1‐type containing 1 (ZNFX1). Both tend to be co‐expressed, although independently regulated [6, 7]. *ZFAS1* is also one of 13 lncRNAs to harbor multiple intronic C/D box small nucleolar RNAs (snoRNAs), a class of RNA involved in ribosomal and small nuclear RNA editing [6, 8]. It was initially identified in murine mammary epithelial cells based on its differential expression during mammary epithelial cell development [6]. According to GTEx, it is abundant and ubiquitously expressed; gene modeling by the Ensembl project predicts that *ZFAS1* may encode up to 47 transcript variants. At least five splice variants of *ZFAS1* have been reported in a breast cancer cell line [7]. Studies on *ZFAS1* deal with the splice variants proximal to ZNFX1, although information on the exact variant studied is often absent or incomplete (e.g., [9, 10, 11, 12]). The role of distal exons, which may arise as a result of alternative splicing, is unknown.

*ZFAS1*, like several lncRNAs, has been linked to cancer [13]. *ZFAS1* is generally considered oncogenic due to its ability to promote cell proliferation and migration, as well as its higher expression in several cancer models, including hepatocellular carcinomas (HCC) [9, 14, 15]. Higher *ZFAS1* expression correlates with a poor prognosis and has been proposed as a potential blood‐derived biomarker for HCC [9, 16]. Its contribution to cancer has been attributed to its capacity to act as a competing endogenous RNA (ceRNA) by sponging miRNAs [9, 10, 11]. The physiological role of *ZFAS1* is largely unclear. *Zfas1* suppression in mouse mammary epithelial cells increased proliferation and metabolic activity, suggesting that it may have antiproliferative activity in non‐transformed models [6]. *ZFAS1* has also been reported in patients suffering from lumbar disk degeneration, where it has been correlated with a pro‐inflammatory profile [17]. In line with a role in inflammation, *ZFAS1* suppression reduces the expression of inflammatory cytokines in a TGF‐β1‐stimulated lung fibroblast cell line [18]. Alternatively, a role for *ZFAS1* in ribosome maturation or activity has been proposed [7]. Zfas1 has also been shown to inhibit the sarcoplasmic reticulum Ca2 + ‐ATPase 2a (Serca2a), leading to calcium overload and mitochondrion‐mediated apoptosis [19]. Zfas1 suppression improved contractile function in a mouse model [12].

Recently, we described the role of the primate‐specific lncRNA *TRIBAL* in regulating hepatocyte function [20]. The expression of several major hepatic regulators was shown to depend on *TRIBAL* expression. In addition to these well‐characterized hepatic regulators, transcription array analysis of *TRIBAL*‐suppressed hepatocytes identified altered expression of hundreds of transcripts of unclear significance. Interestingly, the most impacted transcript was an uncharacterized variant of *ZFAS1*. This work characterizes this variant, as well as other forms of *ZFAS1*, and explores their regulation in the HepG2 hepatoblastoma model, revealing their association with ER stress through the unfolded protein response (UPR), an orchestrated response aimed at restoring homeostasis or ensuring orderly death in irreversibly damaged cells [21].

## Results

### *TRIBAL* suppression is associated with increased *ZFAS1* expression in primary hepatocytes

**Fig. 1:**
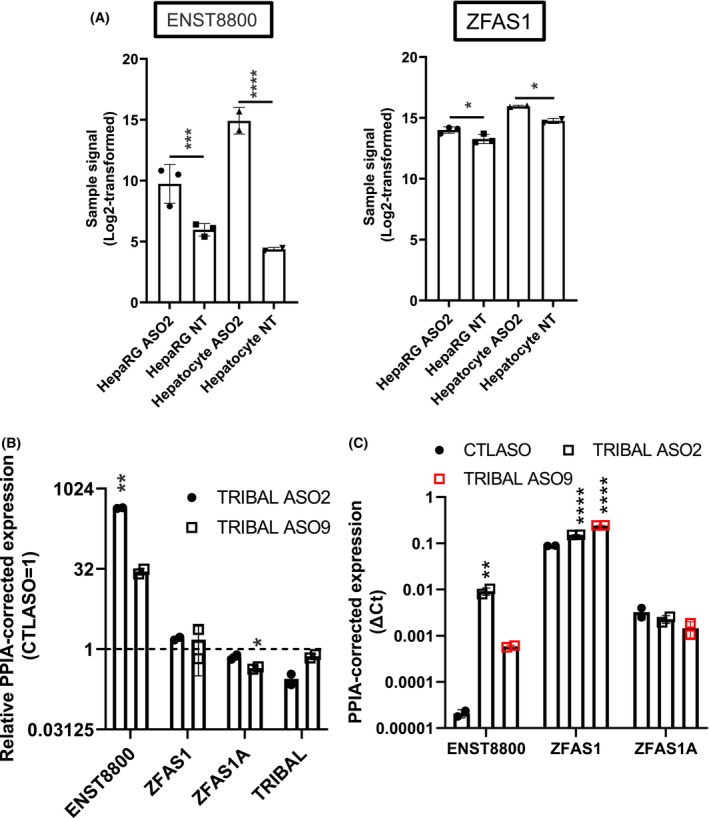
*TRIBAL*‐targeting ASOs upregulate *ZFAS1*. Hepatocytes and HepaRG cells were treated with *TRIBAL*‐targeting gapmer antisense oligonucleotides for 72 h. (A), upregulation of the canonical‐like forms (*ZFAS1*) and *ENST00000618800* (*ENST8800*) measured by microarray analysis in HepaRGs (*n* = 3) and hepatocytes (*n* = 2). Statistical analysis from the Transcriptome Analysis Console (ebayes ANOVA). (B), Validation in 2 additional biological hepatocyte replicates using 2 distinct ASOs by qRT‐PCR and 4 different sets of *ZFAS1*/*ENST8800* PCR primer pairs. Left, Values were normalized to housekeeper *PPIA* expression and are expressed relative to the matching control ASO value. Right, data expressed as 2e‐Ct to illustrate the relative abundance of exon‐exon signals. See Fig. S1 for a schema of the transcripts and positions of the oligonucleotide‐matching regions. In C, ANOVA was performed for each transcript, followed by a Dunnett's *post hoc* test vs. the control (CTLASO). *P < 0.05, **P < 0.01, ***P < 0.001, ****P < 0.0001. Bars represent the mean ± SD, and points correspond to biological replicates.

Following a 72‐h *TRIBAL* suppression in hepatocytes and HepaRG, ClariomD microarray analysis revealed that the most significantly impacted signal (in hepatocytes) corresponded to ‘*ZFAS1*/ENST00000618800’, which exhibited ~ 1500‐fold and 25‐fold upregulation, respectively (Fig. 1A). Although ENST00000618800 is associated with the *ZFAS1* locus in public datasets and the ClariomD microarray, the most abundant ClariomD *ZFAS1* signal matched several putative Ensembl variants, all proximal to the protein‐coding gene *ZNFX1* (Fig. S1). This group of predicted transcripts emerging close to *ZNFX1* and ending before *ENST00000618800* encompasses the previously studied *ZFAS1* variants and will thus be referred to hereafter as *ZFAS1* [6, 7]. The more distal signal spanning *ENST00000618800* exons 1 and 2 will be referred to as *ENST8800*. Whereas *ENST8800* was strongly upregulated, *TRIBAL* suppression had considerably less impact on the more abundant *ZFAS1* signal (Fig. 1A).

### Identification of the *ZFAS1* and *ENST8800* variants in hepatocytes by qRT‐PCR

To substantiate the array evidence, which relies on exon intensity, and delineate the splice variants involved, *ZFAS1* and *ENST8800* abundance were measured by qRT‐PCR following an independent transfection round, using two distinct ASOs and primers spanning the 5′ exons of the predicted *ZFAS1* transcripts. *ENST8800* was again strongly upregulated, although the increase was less pronounced using an ASO targeting a different *TRIBAL* intron, possibly due to less effective suppression (75% for ASO2 vs 25% for ASO9) (Fig. 1B). By contrast, *TRIBAL* ASO treatment elicited a modest upregulation of abundant *ZFAS1* forms, measured using a primer combination spanning exons 2 and 3, which aligns with the array data (Fig. 1B,C and Fig. S1).

In addition, qRT‐PCR detected a low‐abundance transcript spanning *ZFAS1* and *ENST8800* (similar to ENST00000652916 and ENST00000620594 in Fig. S2). However, in contrast *to ENST8800*, its expression was reduced by *TRIBAL* suppression (Fig. 1B,C). This low‐abundance signal, corresponding to a qPCR product comprising *ZFAS1* exon 2 and *ENST8800* exon 2, will be referred to as *ZFAS1*A (for alternate). Note that this constitutes the first experimental support, to our knowledge, of the veracity of this transcript. Although its abundance is considerably lower than *ZFAS1*, it exceeds *ENST8800* under basal conditions. In summary, human hepatocytes express several *ZFAS1* splice variants, which are differently regulated by *TRIBAL* suppression.

### Characterization of splice variant distribution in hepatocytes by PCR and rapid amplification of DNA ends

**Fig. 2:**
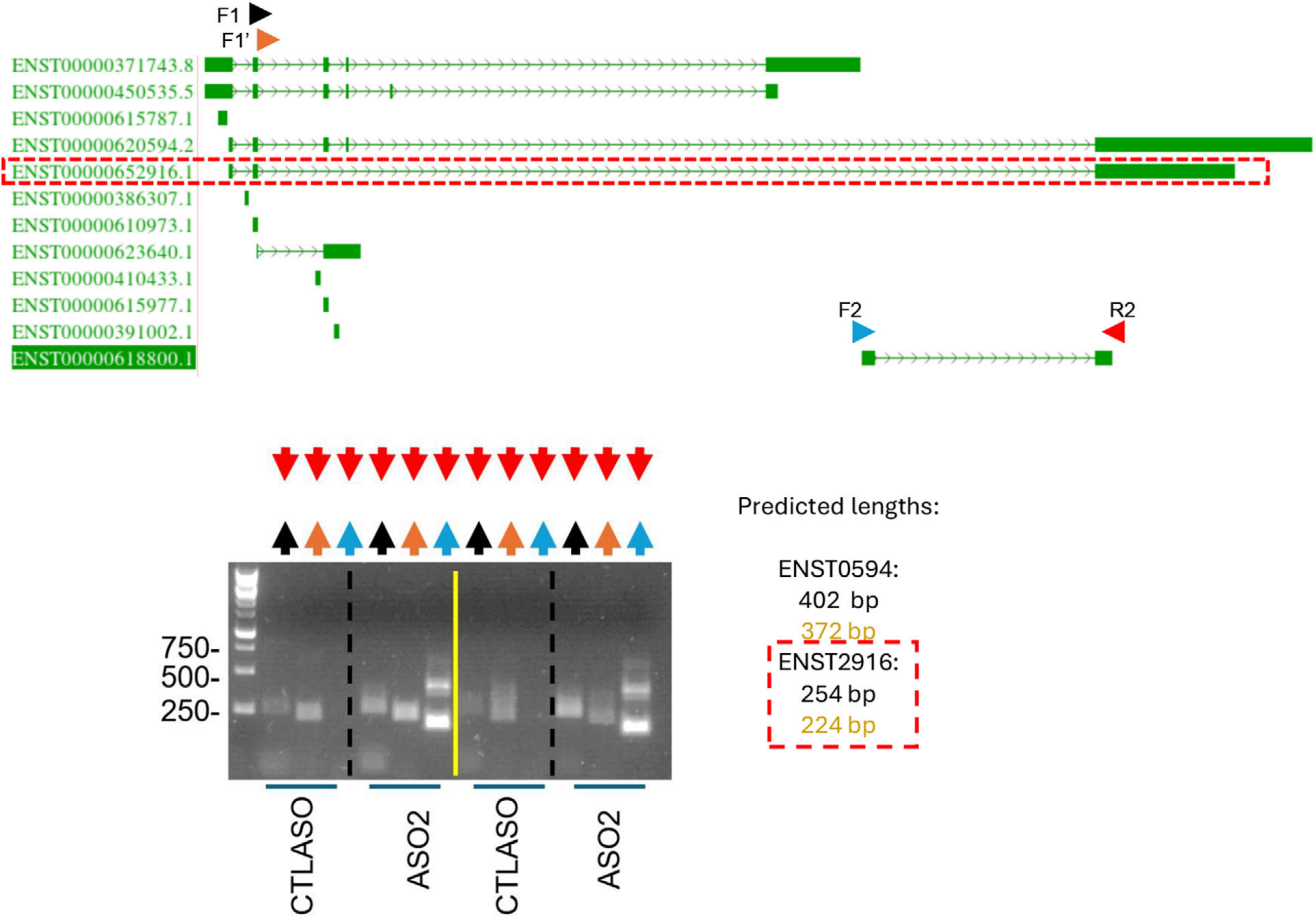
PCR evidence of a basal form spanning *ZFAS1* and *ENST8800* that is consistent with *ENST2916* in primary hepatocytes. Top, schema of *ZFAS1* transcripts adapted from the UCSC genome browser. Primers used are indicated by arrows. A red box highlights *ENST2916*. Bottom, PCR (40 cycles) of cDNA from *TRIBAL* ASO2 and CTLASO‐treated hepatocytes. PCR primers targeting the canonical form exon 2 (F1 and F1′) and the putative terminal exons of the *ZFAS1* and *ENST8800* (R2) are indicated schematically by arrowheads. Samples were loaded on a 1.5% agarose gel. Lanes are color‐coded to indicate the primer pairs used for PCR. Predicted bp lengths based on two Ensembl transcripts (*ENST0594* and *ENST2916*) are shown. Two biological replicates, amplified concurrently, are shown separated by a yellow line.

GTEx data are consistent with lower expression of all forms comprising promoter‐distal exons in all tissues screened, including the liver (Fig. S2). The expression of these forms in hepatocytes was tested by PCR using oligonucleotides that recognized the 3′‐exon and either of the *ZFAS1* qPCR primers (or the *ENST8800*‐specific primer pair as a control). As expected, *TRIBAL* ASO treatment was associated with the appearance of the *ENST8800* signal (Fig. 2). By contrast, primers spanning both forms amplified a product that was present basally and remained essentially unchanged with *TRIBAL* ASO treatment. Interestingly, the size of that PCR product was consistent with the absence of intervening exons (such as ENST00000652916) (Fig. 2). Relevant to the inducible form, the *ENST8800*‐specific PCR amplified, in addition to the expected product, a larger fragment. Restriction digests were consistent with the retention of additional sequences (Fig. S3). To identify *ENST8800* splice variants, rapid amplification of cDNA ends (RACE) reactions were performed on ASO2‐treated hepatocyte to map exons located 5′ of the *ENST8800* exon 2. 3′ RACEs were not performed given that exon 2 of *ENST8800* harbors a consensus polyadenylation signal and that we confirmed that *ENST8800* is polyadenylated; however, the presence of alternatively spliced products cannot be ruled out (Fig. S4). The sequencing of 20 *ENST8800* clones revealed that most forms were consistent with the reported *ENST8800* form (Fig. S5). However, some variants included 1 or 2 additional exons. Moreover, 20/23 forms showed an incomplete *ENST8800* 5′ end, possibly reflecting transcription start site heterogeneity and/or the incapacity of the reverse‐transcriptase to melt the lncRNA structure sufficiently. However, three forms extended further into the 5′ region, consistent with a longer exon 1. Altogether, RACE identified two novel *ENST8800* exons and substantial heterogeneity.

### *ENST8800* suppression does not mitigate the impact of *TRIBAL* suppression on hepatocyte regulators

Based on its strong upregulation in hepatocytes, we hypothesized that increased *ENST8800* expression may play a role in reducing *HNF4A* and *MLXIPL* expression. We focused on these two pivotal transcription factors due to their predicted role in driving transcriptome changes associated with *TRIBAL* suppression [20]. *TRIBAL* suppression was performed in hepatocytes concurrently subjected to 2 pools of *ENST8800* ASOs, each targeting exon 1, the intron, and exon 2 of *ENST8800* (Fig. S6). Upon *TRIBAL* suppression, the inclusion of either pool resulted in substantial interference with *ENST8800* upregulation, whereas minimal basal suppression was observed, suggesting that newly synthesized *ENST8800* was preferentially targeted. Importantly, western blot analysis revealed that *ENST8800* suppression did not significantly affect HNF4A or MLXIPL protein abundance. Thus, the upregulation of *ENST8800* is insufficient to account for changes in HNF4A and MLXIPL abundance.

### Minimal impact of *TRIBAL* suppression on *ZFAS1* in transformed models

Our investigation of the *ENST8800*‐*TRIBAL* relationship was continued in HepG2 and HuH‐7 hepatoma cells, two commonly used hepatocyte surrogate models that have been utilized to investigate the oncogenic contributions of *ZFAS1* [9, 22, 23]. We previously demonstrated that HNF4A, MLXIPL, and other key hepatic regulators were unresponsive to *TRIBAL* ASOs in HepG2 or HuH‐7 cells; however, the response of *ZFAS1* to *TRIBAL* suppression was not examined. Unlike primary hepatocytes, *TRIBAL* suppression in these models had little impact on *ZFAS1* or its variants (Fig. S7A). Nevertheless, the expression pattern and relative abundance of splice variants resembled those of hepatocytes (Fig. S7B and Fig. 1C). Considering prior work linking *ZFAS1* to oncogenesis in HepG2 cells and the greater proximity of HepG2 to primary hepatocytes, our inquiry into *ZFAS1* function was continued in this model.

### *ZFAS1* and *ENST8800* arise from a common transcript that is independently regulated from *SNORD12*

**Fig. 3:**
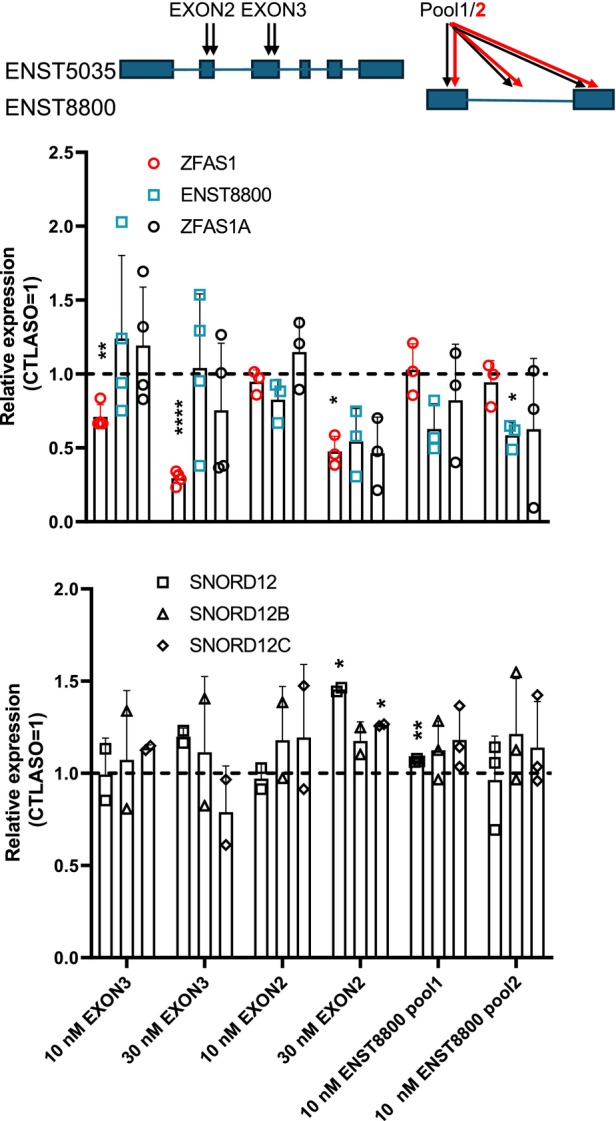
Suppressing exon 2 reduces all forms of *ZFAS1*. HepG2 cells were treated with antisense oligonucleotides for 96 h, and expression of *ZFAS1*, *ENST8800*, and *ZFAS1* was measured by qPCR. Pools of 2 (EXON3 or EXON2) or 3 (pool1 and pool2) were used at the final total oligonucleotide concentration indicated. Top, schematic of the oligo used, matched to *ENST8800* and *ENST5035*. Boxes indicate exons and lines indicate introns. Bottom, quantification of the *ZFAS1* variants (centre; *n* = 3 or 4, as indicated) and *SNORD12* (bottom; *n* = 2 or 3, as indicated) normalized to *PPIA* abundance and expressed relative to the matching CTLASO value. Statistical significance was tested using Student's *t*‐tests vs a value of 1 (CTLASO = 1) in graphpad prism 9. *P < 0.05, **P < 0.01, ***P < 0.001, ****P < 0.0001. Bars represent the mean ± SD, and points correspond to biological replicates.

To better define the exon organization within the *ZFAS1* variants, an investigation leveraging antisense oligonucleotides was initiated. Antisense gapmer oligonucleotides directed toward *ZFAS1* and *ENST8800* sequences were designed and transfected into HepG2 cells. Targeting *ZFAS1* exon 2 reduced all forms of *ZFAS1*, consistent with the inclusion of exon 2 in forms comprising *ENST8800* sequences (Fig. 3A). By contrast, targeting *ZFAS1* exon 3, although highly effective on *ZFAS1*, resulted in a more variable expression of *ENST8800* and *ZFAS1A*, indicating inconsistent retention of exon 3 when distal exons are included. Finally, targeting the *ENST8800* signal using a combination of intronic and exonic sequences reduced its expression by approximately 50% in HepG2 cells, but had no noticeable impact on *ZFAS1*, in line with the considerably lower abundance of the terminal exon forms under basal conditions.

*ZFAS1* harbors three snoRNA genes (*SNORD12, SNORD12B*, and *SNORD12C*), which are situated within consecutive introns. Although the expression of intronic snoRNAs ultimately depends on the transcription of the host gene, their expression is surprisingly uncorrelated [24]. Indeed, the siRNA‐based suppression of *Zfas1* did not alter their abundance in a mouse model [6]. Similarly, ASO‐mediated *ZFAS1* suppression in HepG2 cells did not decrease *SNORD12* levels. Instead, exon 2 suppression resulted in an increased abundance of *SNORD12* and *SNORD12C*, indicating an adaptive response (Fig. 3B). Thus, despite their spatial integration, the SNORD genes and *ZFAS1* are differentially regulated. However, *SNORD12* expression may be increased by reduced *ZFAS1* expression.

### *ZFAS1* and *ENST8800* are cytosol‐enriched

**Fig. 4:**
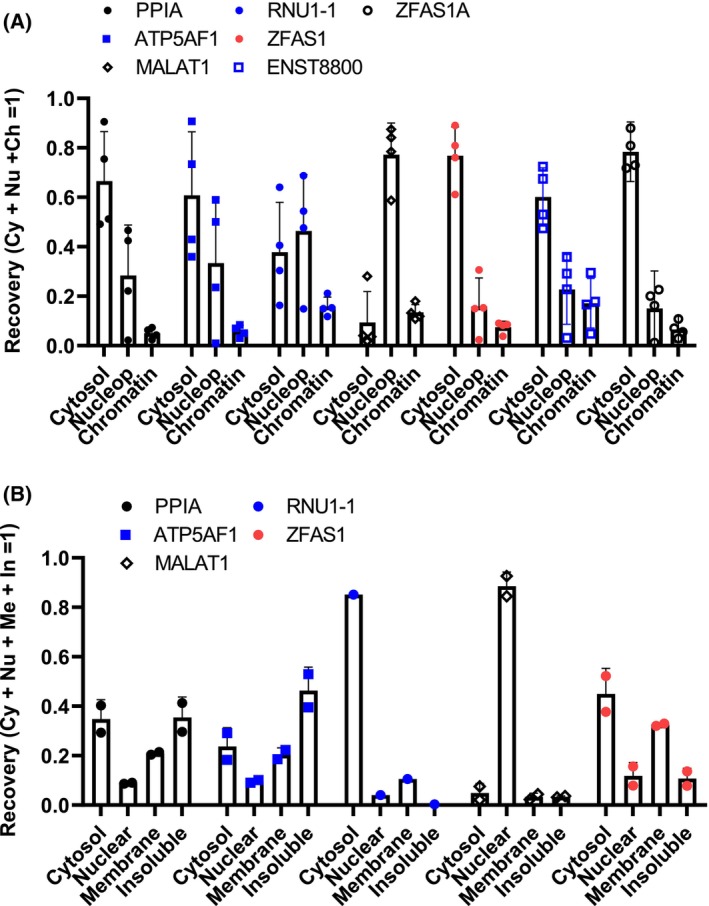
*ZFAS1* variants partition preferentially to the cytosolic compartment in HepG2 cells. (A) Sequential fractionation was performed using incremental stringency to obtain cytosolic, nucleoplasmic, and chromatin‐enriched fractions from HepG2 cells (*n* = 4). (B) Analysis of the RNA‐seq data of Bouvrette *et al*. where HepG2 cells were lysed under hypotonic conditions and fractionated by successive centrifugations and wash steps. Bars represent the mean ± SD, and points correspond to biological replicates.

Location delimits function. Previous work had reported the presence of murine *Zfas1* in nuclear and cytoplasmic fractions, although their relative abundance therein was not quantified [6, 7]. In HFL1 cells, a *ZFAS1*‐reactive signal was detected by *in situ* hybridization in the cytosol, but no *ZFAS1*‐null or suppressed control was provided [18]. The subcellular location of the splice variants was examined in HepG2 cell lysates using incrementally stringent washes. The fractionation of HepG2 cells revealed that the three variants have a similar, predominantly cytoplasmic location, comparable to that of cytosolic mRNAs (Fig. 4A). By contrast, a shuttling small nuclear RNA (snRNA U1) and the nucleus‐enriched lncRNA transcript (*MALAT1*) showed more pan‐cellular and nuclear distributions, respectively. These results demonstrate that all *ZFAS1* variants localize similarly, suggesting that they do not have distinct, compartmentalized functions. To support these findings, fractionation data from alternative, detergent‐free methods were searched in the literature. Although no data were available for individual forms, an RNA‐seq dataset reporting reads mapped to *ZFAS1* in hypotonically‐lysed HepG2 cells was identified [25]. In line with our detergent‐based approach, *ZFAS1* exhibited a predominantly cytosolic distribution and minimal nuclear presence (Fig. 4B). Moreover, a significant fraction of *ZFAS1* was membrane‐associated. These results are consistent with *ZFAS1* playing a predominantly cytoplasmic role, possibly implicating membrane association, which remained to be clarified.

### ER stressors induce *ZFAS1* expression

To yield functional insight, the responsiveness of *ZFAS1* to chemical modulators was examined next. Using a guilt‐by‐association approach, we hypothesized that perturbed expression may inform on *ZFAS1* function. Drugs were selected based on previously described associations of *ZFAS1* with metabolic and apoptotic pathways, which included signaling and metabolic regulators (MAPK kinases (U0126), mTOR (Rapamycin), AMPK (AICAR), etc.) and organelle function modulators (FCCP: mitochondrial uncoupler; thapsigargin and tunicamycin: endoplasmic reticulum (ER) stress). An 18‐h regimen was initially chosen to enable measurable transcriptional changes while reducing potential toxicity confounders associated with longer treatments. Exposure to several compounds increased *ZFAS1* expression (Fig. S8). Uniquely, thapsigargin (Tg) upregulated all the variants, although *ZFAS1* was preferentially increased. A similar pattern was observed with tunicamycin, although only *ZFAS1* reached nominal statistical significance. Moreover, geldanamycin, which causes ER stress by interfering with the chaperone functions of hsp90 and GRP94, also increased *ZFAS1* variants, although nominal significance was not reached [26]. Together, these results suggested that *ZFAS1* is responsive to ER stress induced by protein misfolding.

### Rapid upregulation of *ZFAS1* and UPR effectors in response to thapsigargin

**Fig. 5:**
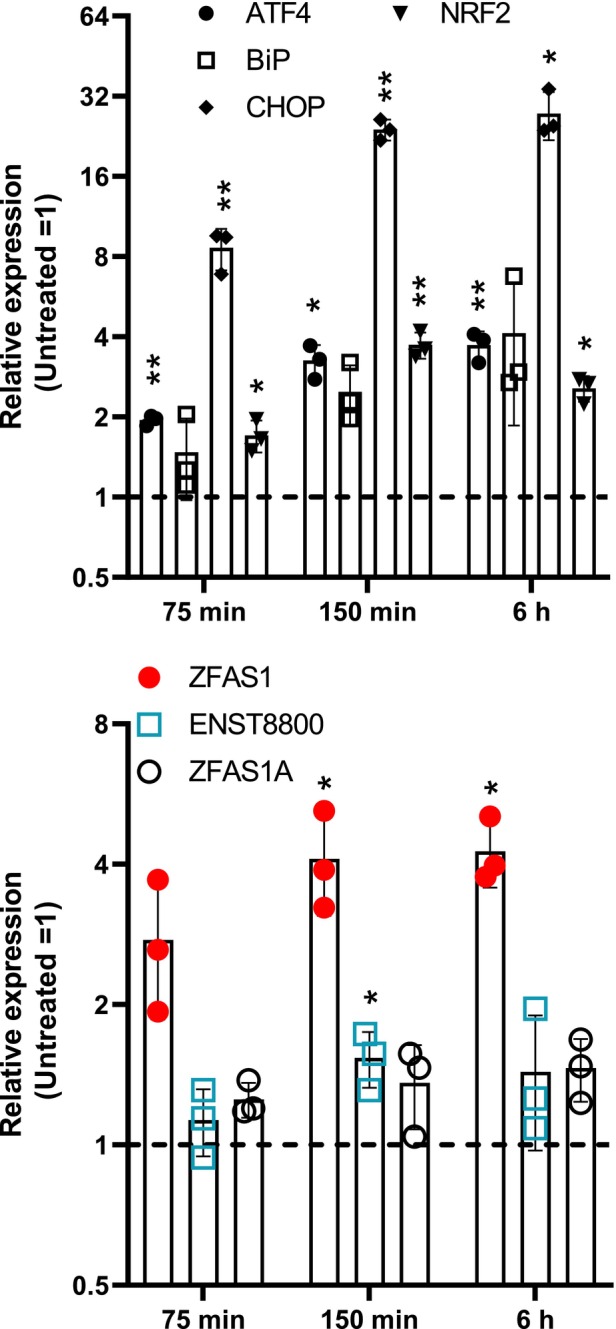
*ZFAS1* upregulation by thapsigargin is rapid. HepG2 cells (*n* = 3) were treated with 1 μm thapsigargin for 75 min to 6 h, RNA was harvested and assayed by qRT‐PCR. Values were normalized to the 0‐min (untreated) time point. Statistical significance was tested using Student's *t*‐tests vs a value of 1 (Untreated = 1) in graphpad prism 9. *P < 0.05, **P < 0.01. Bars represent the mean ± SD, and points correspond to biological replicates.

ER stress associated with protein misfolding is sensed by cells through a coordinated response known as the unfolded protein response (UPR), which includes a central transcriptional component. To confirm UPR activation and examine its timing relative to *ZFAS1* levels, a 6‐h time course was conducted. The UPR typically relies on three ER membrane sensors (PERK, IRE1, and ATF6) and various effectors [21]. Four primary effector transcripts were measured: the transcription factors *CHOP*, *ATF4*, and *NFE2L2*, as well as the chaperone BiP. During ER stress, *CHOP* is upregulated by ATF4 and ATF6, while BiP expression relies on ATF6 during UPR but depends on ATF4 under normal conditions [27]. NFE2L2 is an essential PERK transcriptional effector of the oxidative stress response associated with ER stress [28, 29]. When treated with Tg, all transcripts were significantly increased, with *CHOP* showing the strongest response within 75 min (Fig. 5). Interestingly, *ZFAS1* upregulation followed a similar pattern, showing a threefold increase within 75 min, while *ENST8800* and *ZFAS1A* experienced smaller increases (Fig. 5).

### Inhibition of ER stress signaling curbs *ZFAS1* upregulation but increases *ZFAS1A* expression

**Fig. 6:**
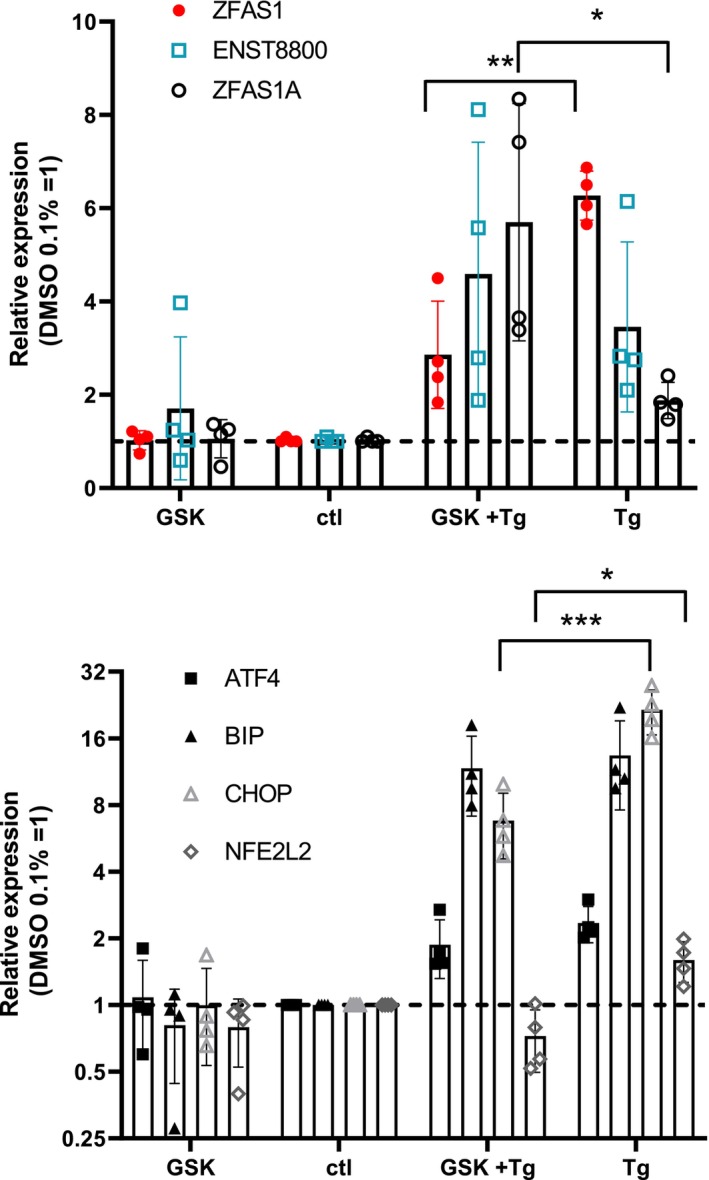
*ZFAS1* variants show contrasting sensitivities to a PERK inhibitor. HepG2 cells treated with GSK2606414 (1 μm), Tg (1 μm), or vehicle for 18 h were analyzed by qRT‐PCR (*n* = 4). In the presence of Tg, all the transcripts were significantly (*P* < 0.05; Student's *t*‐test vs hypothetical value of 1) increased. Statistical significance of GSK vs GSK + Tg treatments was tested using a one‐way ANOVA and multiple comparisons correction (Dunnett's) vs Tg. *P < 0.05, **P < 0.01, ***P < 0.001. Bars represent the mean ± SD, and points correspond to biological replicates.

We hypothesized that PERK played a role in *ZFAS1* upregulation based on the presence of ATF4, one of its main effectors, on the *ZFAS1* promoter in untreated HepG2 cells (Fig. S9). Inclusion of the PERK inhibitor GSK2606414 decreased the Tg‐induced upregulation of *ZFAS1* by approximately 50%, in line with the involvement of PERK (Fig. 6). Interestingly, this effect was isoform‐dependent, as the reduction in *ZFAS1* expression was accompanied by a threefold increase in *ZFAS1A* levels. By comparison, UPR inhibition by GSK2606414 reduced *CHOP* and *NFE2L2* upregulation by approximately 70 and 50%, respectively (Fig. 6).

### NFE2L2 and ATF4 suppression reduce *ZFAS1*

**Fig. 7:**
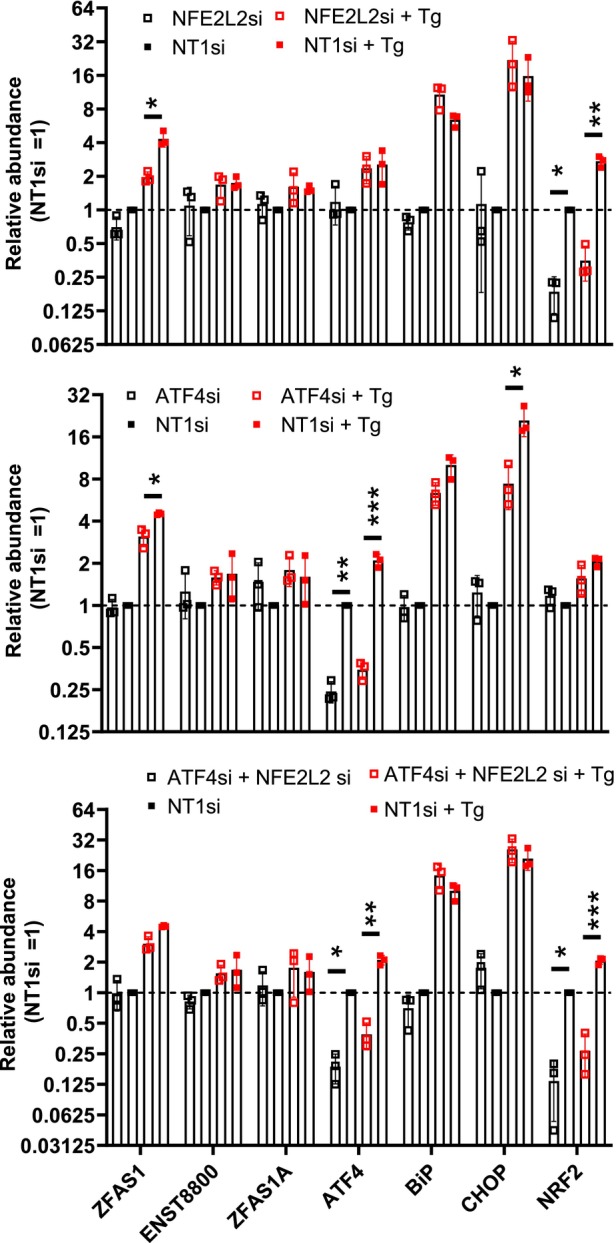
*NFE2L2* and *ATF4* suppression impact the upregulation of *ZFAS1* and UPR players. qRT‐PCR quantification of the indicated transcripts in HepG2 cells treated for 72 h with siRNAs targeting *NFE2L2* (top), *ATF4* (middle), or both (bottom), followed by a six‐hour exposure to 1 μm Tg (*n* = 3 for each panel). Expressed relative to the control (NT1) and vehicle‐treated siRNA values. Student's *t*‐test *P* values are Bonferroni corrected (*n* = 7, *α* = 0.05). *P < 0.05, **P < 0.01, ***P < 0.001. Bars represent the mean ± SD, and points correspond to biological replicates.

Next, the contribution of PERK downstream effectors to *ZFAS1* expression was examined. The levels of *ZFAS1* variants were measured after targeting *ATF4* and *NFE2L2* with their cognate siRNAs. Suppressing either transcription factor for 72 h significantly reduced *ZFAS1* upregulation after a 6‐h Tg treatment, further implicating the UPR in regulating *ZFAS1* (Fig. 7). In contrast, the upregulation of *ENST8800* or *ZFAS1A* after Tg was unaffected by either suppression, suggesting that the low‐abundance transcripts are regulated differently. A combined siRNA approach targeting both transcription factors did not further reduce *ZFAS1* upregulation, suggesting epistatic rather than independent contributions.

### *ZFAS1* suppression has minimal impact on the expression of UPR mediators

**Fig. 8:**
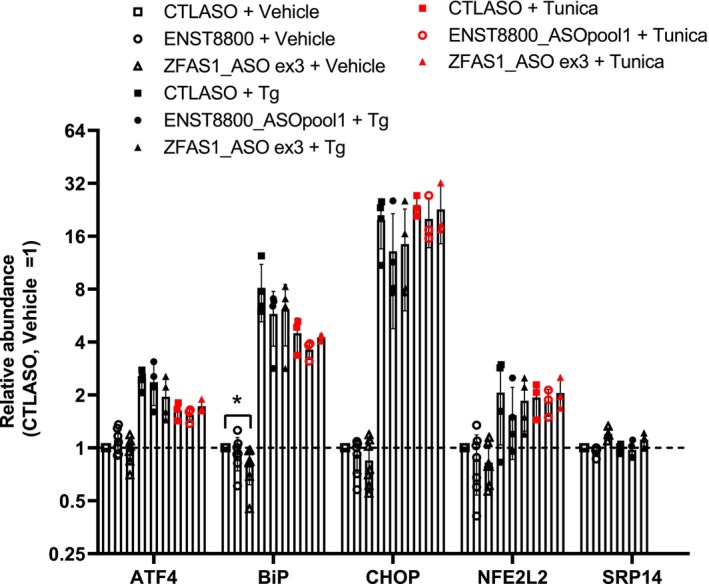
Impact of *ZFAS1* and *ENST880*0 suppression on the expression of UPR mediators. qRT‐PCR analysis of the indicated transcripts in HepG2 cells. *ZFAS1* variants were suppressed for 72 h, followed by a 6 h Tg (1 μm) or tunicamycin (6 μm) treatment. Both sets of experiments (*n* = 4 for each set) were performed independently, and control (vehicle) values were combined. The *SRP14* values were obtained from the Tg‐treated samples. Statistical significance was tested using Student's *t*‐tests vs a value of 1 (CTLASO, vehicle = 1) in graphpad prism 9. Bars represent the mean ± SD, and points correspond to biological replicates.

Given the rapid upregulation of *ZFAS1* in response to ER stress, the possibility that *ZFAS1* might in turn influence the expression of UPR mediators was considered. The levels of UPR effectors, both at baseline and after treatment with Tg or tunicamycin, were measured in cells with *ZFAS1* or *ENST8800* suppression. Whereas no impact was noticed in the presence of ER stressors, *ZFAS1‐*suppressed cells exhibited lower levels of *BiP* under basal conditions (Fig. 8). By contrast, levels of the alternative housekeeper transcript *SRP14* remained unchanged, thus excluding the possibility that reductions were due to increased expression of the normalization transcript. However, protein levels were not significantly affected, as assessed by western blotting, suggesting that *ZFAS1* is dispensable for the UPR (Fig. S10).

### *ZFAS1* suppression reduces HepG2 cell viability basally and in response to Tg

**Fig. 9:**
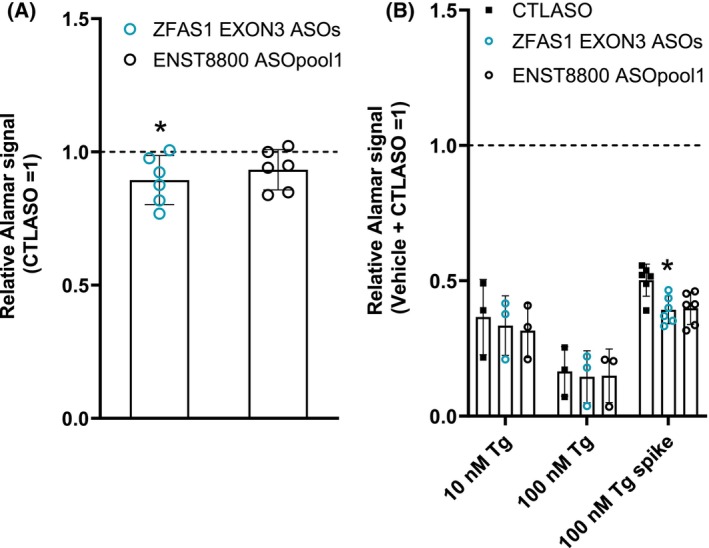
*ZFAS1* suppression reduces cell viability. Alamar blue viability assay of HepG2 cells. (A) cells were treated continuously with *ZFAS1* ASOs for 96 h before the assay (*n* = 6). (B) cells were treated for 48 h with ASOs, followed by a 48 h thapsigargin treatment or for 72 h with the ASOs, followed by a 1 h Tg spike and a 48 h wash‐out period (Tg, *n* = 3; Tg spike, *n* = 6). *P < 0.05. Statistical significance was tested using Student's *t*‐tests vs a value of 1 (CTLASO or Vehicle + CTLASO = 1) in graphpad prism 9. Bars represent the mean ± SD, and points correspond to biological replicates.

We hypothesized that changes in *ZFAS1* levels in response to Tg might affect cell viability and survival. Previous studies have shown inconsistent results regarding the role of *ZFAS1* in hepatoma proliferation. A fivefold increase in *ZFAS1* expression (though the specific variant was not indicated) was shown to enhance the proliferation of HepG2 cells [9]. Moreover, *ZFAS1* knockdown did not affect the proliferation of HepG2 and Huh‐7 cells but was linked to reduced invasion in transwell assays [9]. However, another study found that silencing *ZFAS1* led to decreased colony formation and proliferation in HepG2 cells [14]. The involvement of *ENST8800* in cell proliferation was not examined. To assess whether *ZFAS1* variants are necessary for HepG2 cell proliferation and viability, cells were treated for 96 h with ASOs targeting *ENST8800* and *ZFAS1* exon 3. Targeting *ZFAS1* or *ENST8800* reduced viability by approximately 10%, as measured using an Alamar assay, with statistical significance observed only for *ZFAS1* targeting (Fig. 9A).

Next, the impact of Tg treatment combined with *ZFAS1* suppression was examined. Initially, a continuous treatment approach was used. A dose–response curve showed that Tg was highly toxic over 48 h (Fig. S11), as expected. Interestingly, all forms of *ZFAS1* remained highly upregulated after 48 h of Tg exposure. To evaluate the roles of *ZFAS1* variants during significant but not maximal cell death, experiments were conducted at 10 and 100 nm Tg. Under these conditions, *ZFAS1* suppression did not influence cell survival. We hypothesized that constant stress might be too severe to assess physiological contributions. The experiment was repeated with a 1‐h pulse of 100 nm Tg followed by a 48‐h recovery period. This approach revealed a requirement for *ZFAS1* to support maximal viability (Fig. 9B).

## Discussion

Here, we provide novel information on the role of and regulation of *ZFAS1* in primary and transformed hepatocytes. We first characterize multiple *ZFAS1* variants and test the impact of *ENST8800* suppression on the hepatocyte response to *TRIBAL* suppression. This was motivated by the observation that *ENST8800* is robustly upregulated in response to *TRIBAL* suppression in hepatocytes. We report that preventing *ENST8800* upregulation did not alter the response of hepatocytes to *TRIBAL* suppression, as assessed by similar reductions in HNF4A and MLXIPL. Importantly, since we were primarily interested in elucidating the mechanisms linking these two major regulators and *TRIBAL*, the global impacts of *ZFAS1* suppression in hepatocytes were not examined.

Early in this investigation, we realized that the *ZFAS1* transcripts, particularly the low‐abundance forms, were largely uncharacterized. To fill this knowledge gap, we focused our attention on HepG2 cells, which have been previously utilized to study *ZFAS1* in the context of oncogenesis. HepG2 is, in this regard, a model of convenience, allowing for the rapid and cost‐effective exploration of *ZFAS1*, albeit with limitations inherent to immortalized cell lines. One shortcoming, also observed in HuH‐7, is the loss of the *TRIBAL‐ZFAS1* functional relationship that is present in primary hepatocytes. A key rationale supporting the use of the HepG2 model to yield generalizable and informative findings is the ubiquitous expression of *ZFAS1*, as indicated by public datasets (e.g., GTEx, BioGPS), consistent with a fundamental biological role.

We demonstrate that all *ZFAS1* variants are cytoplasm‐enriched lncRNAs that respond to UPR activation in HepG2 cells. Of particular interest to liver disease, the UPR is impaired in several liver pathologies, including fatty liver disease, viral hepatitis, and alcohol‐induced liver injury [30]. Moreover, UPR activation protects against hepatic steatosis [31]. Treatment with either Tg or tunicamycin, two classical UPR inducers, was sufficient to rapidly and stably increase *ZFAS1* variants, with the most abundant form being the most responsive. Additionally, *NFE2L2* and *ATF4* were necessary for the upregulation of the abundant *ZFAS1* form. As the combined *NFE2L2/ATF4* suppression did not further reduce *ZFAS1* expression, they may operate as heterodimers, as previously reported [32]. Incomplete blockage of *ZFAS1* and upregulation of UPR effectors may be due to the presence of residual ATF4 and NFE2L2. Alternatively, additional pathways may be implicated, consistent with the similarly incomplete impact of PERK inhibition. These could include other branches of the UPR, as PERK's role is not limited to ATF4 activation, or be non‐PERK‐mediated since the PERK inhibitor GSK2606414 can inhibit other kinases (RIPK1 and KIT1) [33, 34, 35].

Significantly, the lower abundance splice variants were less responsive to Tg and were less affected by *NFE2L2* and *ATF4* suppression. Moreover, PERK inhibition potentiated the Tg‐induced upregulation of *ZFAS1A*. Interestingly, GSK2606414 has recently been shown to activate GCN2, suggesting that GCN2 or another unidentified kinase may promote the expression of these *ZFAS1* splice variants [36]. Beyond revealing contrasting expression profiles, this leaves open the possibility that forms containing the terminal exons may exert different functions. Indeed, these terminal forms are probably diverse, as suggested by *TRIBAL* suppression in primary hepatocytes, which was associated with increased *ENST8800* and *ZFAS1* but reduced *ZFAS1A*. Clarifying their roles will require the expression of individual splice variants, contingent upon further exploration and delineation of the *ZFAS1* repertoire.

While responsive to the UPR, the role of *ZFAS1* therein, if any, is less clear. Testing for the abundance of UPR effectors, *ZFAS1* suppression reduced *BiP* expression only under steady‐state conditions, although the effect was small and did not reach statistical significance at the protein level. However, the UPR is an orchestrated response, and the expression levels of four effectors cannot fully capture it; further experiments will be needed to properly test *ZFAS1*'s involvement [37]. For instance, impacts on the core initiators (ATF6, PERK, and IRE1) were not examined. The slightly reduced viability of *ZFAS1*‐suppressed cells in response to the acute Tg treatment is certainly consistent with a protective role, although not necessarily via the UPR.

Although we focused on the UPR, motivated by the effects of ER function disruptors, several other inhibitors also increased levels of *ZFAS1* and its variants. While strict nominal significance was not always achieved due to a combination of small effect sizes and low power, upregulation was concordant across variants, suggesting concerted regulation. In line with the close integration of mitochondrial and ER compartments, *ZFAS1* variants were upregulated by the mitochondrial uncoupler FCCP, which may further link *ZFAS1* to ER dysfunction [38]. Note that in the absence of changes (e.g., AICAR), the lack of positive control limits conclusions. Moreover, inhibitory transcriptional effects may not be adequately captured, given the exceptional stability of *ZFAS1*, which far exceeds the median lncRNA stability of approximately 3–4 h [39]. Indeed, we demonstrate that levels of *ZFAS1* and its variants were resistant to an 18‐h treatment with the Pol II inhibitor ACTD; treatment with the Pol II and CK2 inhibitor DRB in fact showed a trend toward increased abundance. Work in murine models reported that *Zfas1* levels remain unchanged and may even increase slightly in cells treated with ACTD for up to 32 h [6, 39]. Highly stable protein‐coding transcripts preferentially encode proteins playing constitutive roles, such as those involved in basic metabolic processes [39, 40]. Extrapolating these findings to lncRNAs supports the notion that *ZFAS1* exerts housekeeping functions.

One plausible role of *ZFAS1* relates to ER calcium regulation. Zfas1 has been shown to interact with Serca2a, a regulator of sarcoplasmic calcium levels in cardiac muscle [12, 19]. Although SERCA2A is muscle‐specific and sarcoplasmic, the alternatively spliced SERCA2B is an ubiquitously expressed ER calcium pump that plays an essential role in the liver [41, 42]. Since both variants share the same *ZFAS1* interaction domain, *ZFAS1* may interact with SERCA2B to ensure calcium homeostasis within the ER. Alternatively, given the presence of three intronic snoRNAs within *ZFAS1* and its association with ribosomes, it may regulate translation or ribosome maturation, as previously suggested [7, 24, 43]. Specifically, *ZFAS1* could regulate the translation arm of the UPR, which involves inhibition of global translation and the enhanced translation of specific transcripts [21]. Given the functional integration of the rough ER and the translation machinery, *ZFAS1* may modulate translation efficiency or selectivity through its ER association. Future studies will investigate these possibilities.

## Methods

### Cell culture

Growth conditions were previously described [44]. Briefly, primary human hepatocytes (GIBCO (Thermo Fisher), Waltham, MA, USA; Cryo Human Hepatocytes LOTHU8413) and HepaRG cells (CVCL_9720), obtained from Biopredic International, were grown in William's E‐based media with supplements. HuH‐7 (CVCL_0336) and HepG2 (CVCL_0027) cells, obtained from the JCRB Cell Bank and the ATCC, respectively, were maintained in LG‐DMEM supplemented with fetal bovine serum (10% final) and penicillin/streptomycin and were passaged at ratios of 1 : 3 (HuH‐7) or 1 : 4 (HepG2) every 3–4 days. Cell lines were verified to be free of mycoplasma contamination by PCR and microscopic examination. Cell authentication was performed by the supplier; cell identity, as determined in‐house by expression profile and morphology, was consistent with the published literature and the supplier's information. With the following exception, drugs used were obtained from Cayman Chemical: FCCP (Thermo Fisher Scientific, Waltham, MA, USA), Wortmannin (Cell Signaling, Danvers, MA, USA), and PMA (MedChemExpress, Monmouth Junction, NJ, USA).

### Viability assay

Viability was assessed using the resazurin‐based Alamar HS assay (Thermo Fisher Scientific). Fresh media containing a 1 : 10 dilution of Alamar HS was added to the cells for 30 min, and fluorescence was measured using a Bio‐Tek plate reader at nm. Duplicate values were measured, and a cell‐free control was subtracted from the readings to account for background noise. The data were normalized to the control ASO‐treated sample values.

### Antisense gapmer oligonucleotides

Antisense oligonucleotides were designed as gapmers and obtained from Integrated DNA Technologies (IDT); sequences are listed in Supporting Information S1. Primary hepatocytes and HepaRG transfections were described previously [20, 44]. HepG2 cells were split at a density of 100 000 cells per well (24‐well plates) and transfected immediately with the ASOs or siRNA. ASO Transfections were initially performed using 10 or 30 pmol per well and 1 μL of Lipofectamine iMAX per well (using 100 μL of Optimem). This was later increased to 30 pmol per well (and 1 μL of Lipofectamine iMAX) to increase suppression efficiency. For siRNA experiments, 10 pmol and 1 μL of Lipofectamine iMAX were used per well. For dual silencing, 10 pmol of each siRNA was combined with 1 μL of Lipofectamine iMAX per well.

### RNA isolation and qRT‐PCR

RNA purification, cDNA synthesis, and quantitative real‐time PCR (qRT‐PCR) were performed as described previously [45]. PPIA, which was found to be consistently unchanged in response to ASO treatment in transcription arrays, was used as a housekeeping gene for normalization using the Δ*C*_t_ method. The *ZFAS1* qPCR primer pairs had comparable amplification efficiencies (> 95%) as determined by serial dilutions of hepatocyte cDNA. Oligonucleotide sequences are listed in Supporting Information S1. qRT‐PCR was performed using 1 μL of cDNA in 25 μL reactions for 40 cycles with Terra PCR Direct Polymerase (Takara Bio, San Jose, CA, USA).

### Western blotting

Cell lysates were obtained by in‐well lysing of rinsed cells for 5 min on ice in lysis buffer (50 mm Tris–HCl, 150 mm NaCl, 1% Triton X‐100, pH 7.4) supplemented with protease and kinase inhibitors (PhoStop and Complete, Roche, Mississauga, ON, Canada), followed by centrifugation (2 min at 16 kg). Supernatants were denatured in 1X Laemmli SDS/PAGE buffer (95 °C) for 5 min and then subjected to SDS/PAGE (8% gels). Gels were transferred (15% Methanol in 1X transfer buffer) to nitrocellulose membranes (30 V for 16 h). Even loading and transfer were confirmed by Ponceau staining. Blots were then destained and blocked for 1 h in Intercept buffer (LI‐COR). Detection was performed using antibodies diluted in PBS/0.1% Tween or TBS/0.1% Tween (for UPR effectors). Blots were incubated for 16 h with primary antibodies (1 : 1000 dilution) and 1 h with secondary antibodies (Donkey anti‐mouse IRdye680RD and Donkey anti‐rabbit IRdye800CW; LI‐COR) diluted at 1 : 20 000. Antibodies are detailed in Supporting Information S1. Four 30‐s washes in PBS (or TBS, for UPR effectors) were performed after each antibody incubation. Blots were imaged on a ChemiDoc MP imaging system (BioRad, Hercules, CA, USA). All images were within the instrument's dynamic range and were only adjusted in contrast and intensity.

### Cell fractionation

HepG2 lysates were fractionated sequentially into cytosolic (low salt extract), nucleoplasmic (defined as extractable with 1 m Urea and 0.3 m NaCl), and an insoluble residual fraction (chromatin‐enriched). One well of a confluent 6‐well plate was briefly rinsed in ice‐cold PBS, scraped, and centrifuged (1 min, 500× ***g***, 4 °C). Pellets were then resuspended in 100 μL PBS by gentle pipetting and mixed with four volumes of hypotonic lysis buffer (HLB) (0.3 M sucrose, 10 mm Tris–HCl, 5 mm MgCl2, 1% Triton X‐100, pH 7.5) containing 0.25% (V/V) Protector RNAse Inhibitor (Roche) for 5 min on ice. Samples were then centrifuged (2 min, 2000× ***g***, 4 °C), and the supernatant was recovered as the cytoplasmic fraction. An aliquot (20%) was diluted in three volumes of TriPure reagent for isolation. The pellet was resuspended in 100 μL HLB containing 0.3 m NaCl and 1 m Urea for 5 min on ice. Following centrifugation (2 min, 2000× ***g***, 4 °C), the supernatant (nuclear fraction) was recovered and diluted in three volumes of TriPure reagent. The remaining pellet was briefly resuspended in 100 μL HLB, and TriPure reagent was immediately added to the suspension (chromatin fraction). All samples were frozen at −80 °C before RNA isolation using Direct‐Zol RNA Miniprep Kits (Zymo Research, Irvin, CA, USA).

For the analysis of the supplemental dataset of Bouvrette *et al*., reads within each fraction were normalized to total fragments per kilobase per million mapped reads (fkpm) for each transcript. ZFAS1 corresponds to ENSG00000177410. When available, the average of two technical replicates was used; otherwise, a single technical replicate was used. Values shown represent the means of two values obtained following alternate distinct enrichment procedures: rRNA depletion and polyA enrichment. For *U1* (*RNU1‐1*), complete data from a single set were available and are shown.

### Rapid amplification of DNA ends

This 5′ RACE protocol was performed using a template‐switching approach using the template‐switching RT enzyme mix and associated protocol (New England Biolabs, Ipswich, MA, USA). Briefly, ASO2‐treated hepatocyte RNA was converted to cDNA using an oligo(dT) primer and a template‐switching oligo (see Supporting Information S1 (for sequences)). The cDNA was then subjected to PCR using a Terra Direct PCR mix (Takara Bio). Two nested PCR rounds (35 cycles each) were performed using gene‐specific and TSO‐specific primers; enrichment of the correct product was ascertained by qRT‐PCR using primer sets targeting *ENST8800*. A final round of 15 cycles with FastStart Taq (Roche) was performed using the inner primers, and the resulting PCR product was purified using a spin column and cloned using TOPO‐TA cloning (ThermoFisher). Sanger sequencing was performed on 24 positive clones (as determined by *ENST8800* qPCR), 23 of which were successful.

### Statistical analysis

Statistical testing was performed assuming normal distribution, using ANOVA for comparing multiple groups or, for control‐normalized values, Student's 2‐tailed *t*‐test against a control value of 1 in Prism 9. Multiple‐hypothesis corrections of ANOVA tests were made with Dunnett's. Given the exploratory nature of this work (no *a priori* expected variance), no power‐based sample size estimates were performed; rather, 3–4 independent biological repeats were performed to gauge biological variability. All data points shown represent biological replicates, and error bars indicate standard deviations.

## Conflict of interest

The authors declare no conflict of interest.

## Author contributions

SS designed, performed, analyzed experiments, and wrote the manuscript. PL provided technical assistance, and RM acquired funding and edited the manuscript.

## Supporting information


**Fig. S1.** Basic Gene Annotation Set of *ZFAS1* forms from GENCODE visualized via the UCSC gene browser with the location of primers used for qRT‐PCR.
**Fig. S2.** *ZFAS1* forms indexed in the GTEx Database.
**Fig. S3.** MscI digest of the ENST8800‐specific PCR product.
**Fig. S4.** *ENST8800* and *ZFAS1* variants are polyadenylated in primary hepatocytes.
**Fig. S5.** Map of transcripts identified by 3′ RACE.
**Fig. S6.** Preventing *ENST8800* upregulation does not mitigate the impact of *TRIBAL* suppression.
**Fig. S7.** Minimal impact of TRIBAL suppression on ZFAS1 expression in HuH‐7 and HepG2.
**Fig. S8.** *ZFAS1* abundance in response to a panel of modulators.
**Fig. S9.** ATF4 is present on the *ZFAS1* promoter region and proximal to *ENST8800*.
**Fig. S10.** No significant impact of ZFAS1 suppression on the UPR effectors.
**Fig. S11.** Thapsigargin is toxic and increases ZFAS1 durably.

**Supporting Information S1.** List of nucleotides, antibodies used, and novel ENST8800 sequences.

**Data S1.** Uncropped blots. Complete versions of the blots shown in the manuscript.

## Data Availability

Data supporting the findings in this study are available from the corresponding author (ssoubeyrand@ottawaheart.ca). This study uses publicly available RNA‐seq data from the Supplemental Data of Bouvrette et al. [25]. Hepatocyte and HepaRG expression array data are available at the Gene Expression Omnibus https://www.ncbi.nlm.nih.gov/geo/ (GSE248931 and GSE284599, respectively).
