# Supplementary material for: Differential regulation of ZFAS1 splice variants by endoplasmic reticulum stress in hepatocyte cell lines
Source: FEBS Open Bio. 2026 Feb 6;16(6):1115–31. doi: 10.1002/2211-5463.70185 (PMC13238724; doi:10.1002/2211-5463.70185)
Supplement: Supplementary file 1 — Fig. S1. Basic Gene Annotation Set of ZFAS1 forms from GENCODE visualized via the UCSC gene browser with the location of primers used for qRT‐PCR. Fig. S2. ZFAS1 forms indexed in the GTEx Database. Fig. S3. MscI digest of the ENST8800‐specific PCR product. Fig. S4. ENST8800 and ZFAS1 variants are polyadenylated in primary hepatocytes. Fig. S5. Map of transcripts identified by 3′ RACE. Fig. S6. Preventing ENST8800 upregulation does not mitigate the impact of TRIBAL suppression. Fig. S7. Minimal impact of TRIBAL suppression on ZFAS1 expression in HuH‐7 and HepG2. Fig. S8. ZFAS1 abundance in response to a panel of modulators. Fig. S9. ATF4 is present on the ZFAS1 promoter region and proximal to ENST8800. Fig. S10. No significant impact of ZFAS1 suppression on the UPR effectors. Fig. S11. Thapsigargin is toxic and increases ZFAS1 durably. [file FEB4-16-1115-s003.pdf]

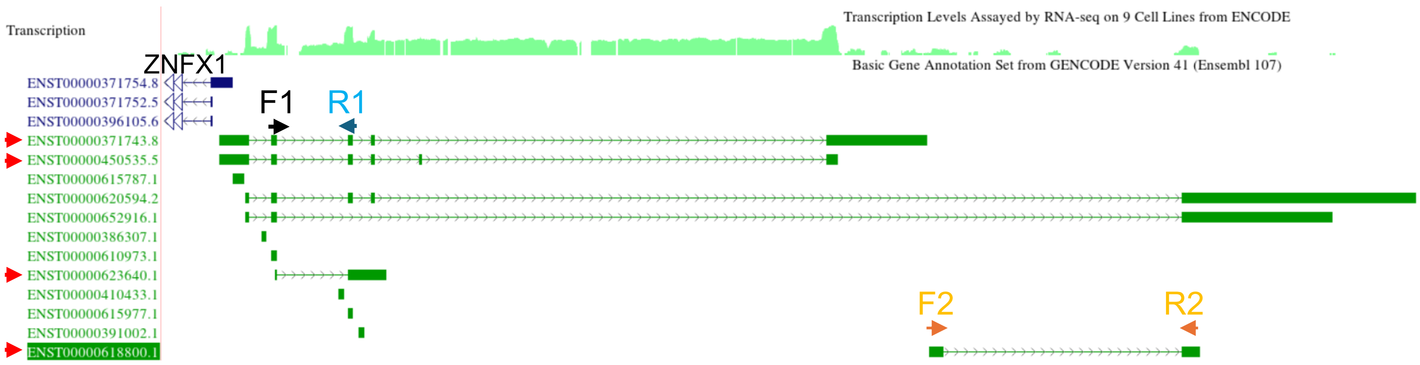

Figure S1. Basic Gene Annotation Set of *ZFAS1* forms from GENCODE visualized via the UCSC gene browser with the location of primers used for qRT-PCR. Transcription levels from the hepatoblastoma cell line HepG2 are shown on top to illustrate the contrasting expression profile mapping to the *ZFAS1* region. The cyan arrow indicates the position of the reverse PCR primer R1 used to quantify *ZFAS1*. Orange arrows indicate the approximate positions of the *ENST8800* PCR primers (F2 and R2). *ZFAS1A* was quantified with the F1 and R2 primers. Red arrowheads indicate the *ZFAS1* models represented on the ClariomD array.

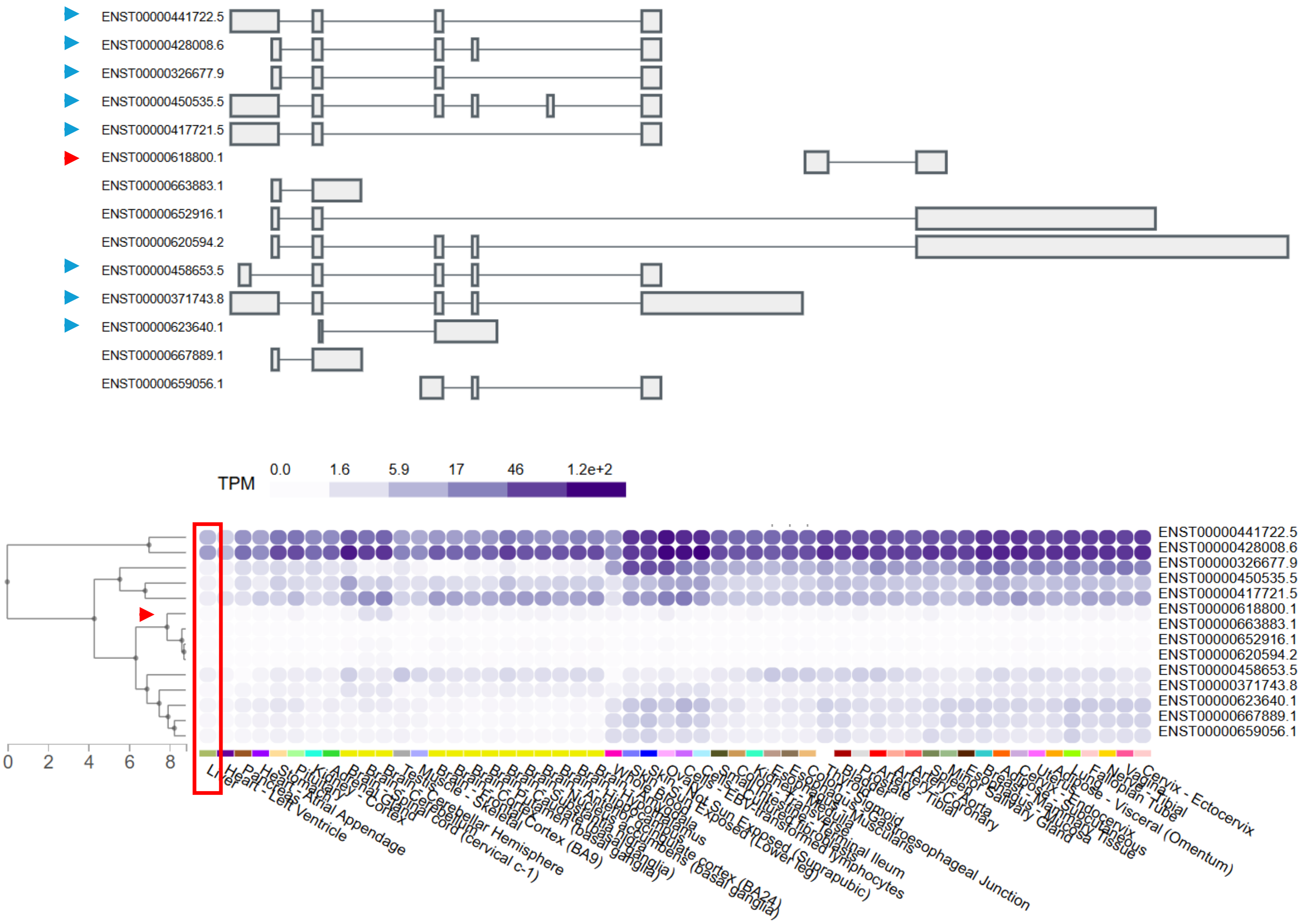

Figure S2. *ZFAS1* forms indexed in the GTEx Database. Top, various splice variants inferred from RNAseq and array data leveraged by GTEx. Bottom, expression of these variants in the GTEx tissue panel in transcripts per million (TPM). *ENST8800* expression, indicated by a red arrow, is significantly lower than most *ZNFX1*-proximal forms. Cyan arrows point to transcripts measured (in aggregate) by the ClariomD array. Liver values are boxed in red. Adapted from https://www.gtexportal.org/home/gene/ZFAS1.

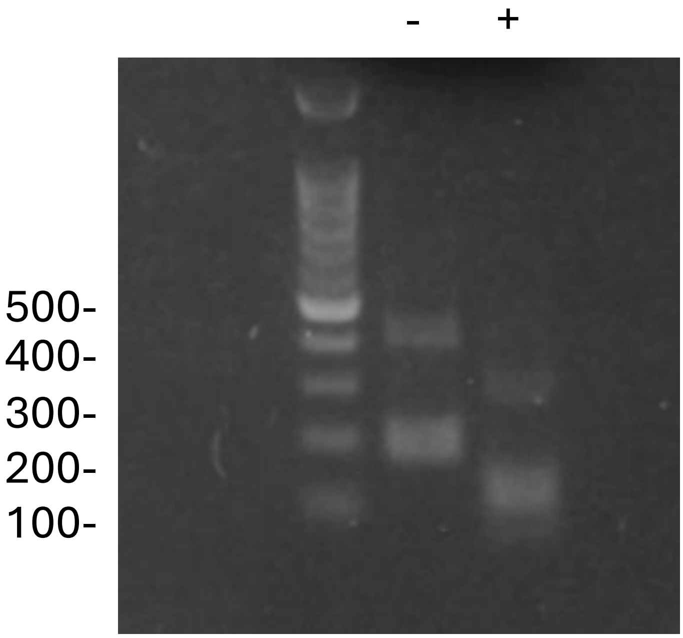

Figure S3. **MscI digest of the *ENST8800*-specific PCR product**. The ~190 bp *ENST8800* PCR product amplified from hepatocytes is predicted to include a MscI site, resulting in 118 and 71 bp products upon cleavage. A ~420 bp product derived from *ENST8800* (with no additional MscI site) is predicted to yield ~ 300 bp and 118 bp. Restriction digest results in a reduction of the ~420 and ~190 bp products and the appearance of bands consistent with the inclusion of additional sequences in a subset of the PCR population.

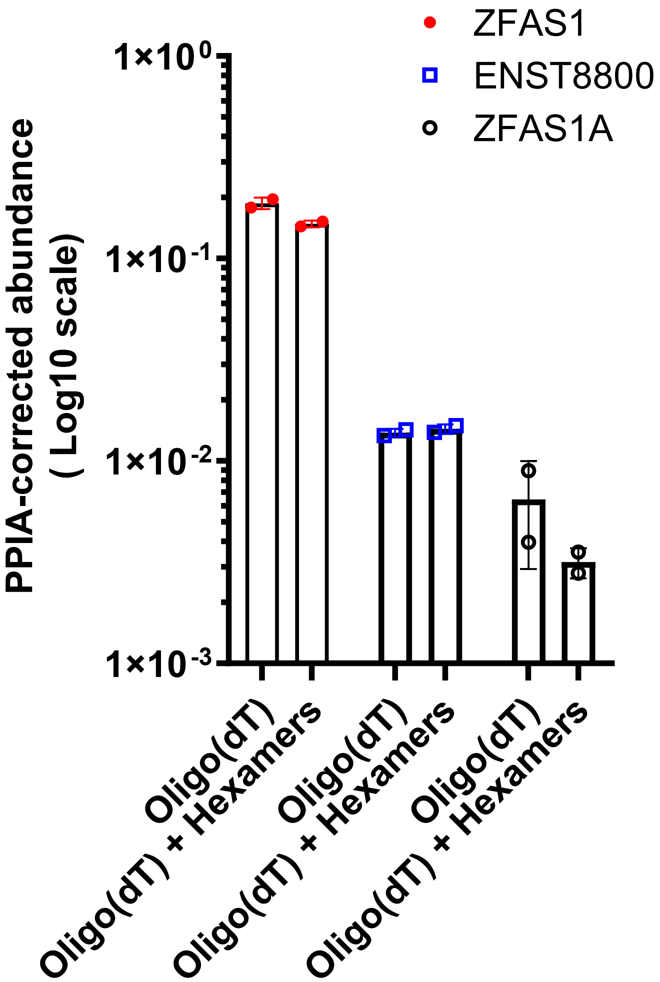

Figure S4. ***ENST8800* and *ZFAS1* variants are polyadenylated in primary hepatocytes**. RT-qPCR was performed on cDNAs prepared using either oligodT oligonucleotides alone or oligodT oligonucleotides together with random hexamers (standard condition). The RNA was from ASO2-treated hepatocytes. Both approaches were indistinguishable (Student's t-test). The average of 2 biological replicates is shown.

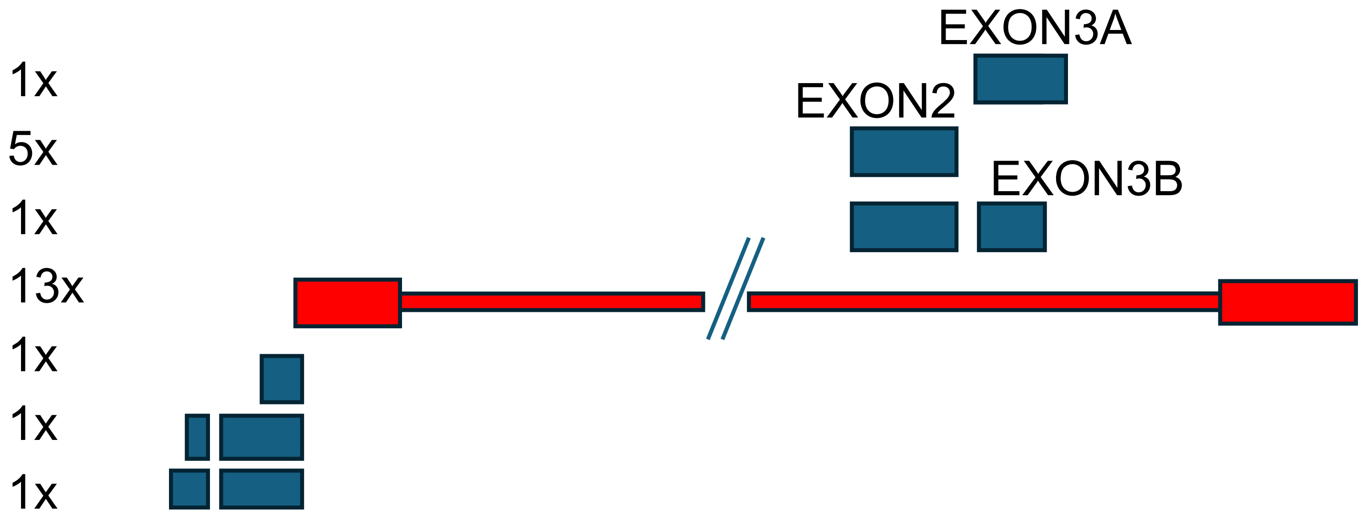

Figure S5. **Map of transcripts identified by 3' RACE**. Exon sizes are drawn approximately to scale. The *ENST8800* form is shown in red. Sequences are included **as Supplemental information**.

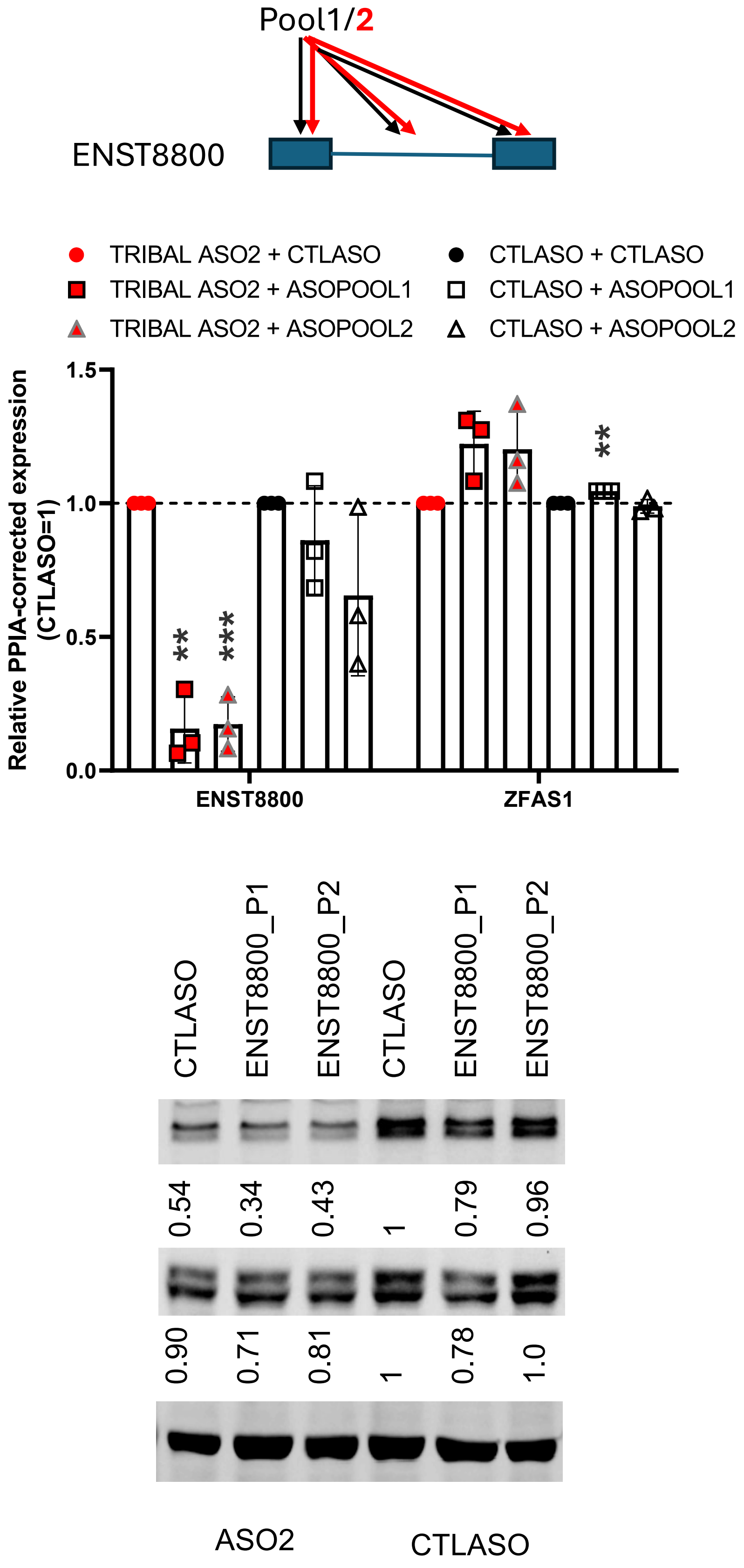

Figure S6. Preventing *ENST8800* upregulation does not mitigate the impact of *TRIBAL* suppression on MLXIPL and HNF4A. Hepatocytes were treated for 48 h with the indicated 1:1 mixture of *TRIBAL* and *ZFSA1* ASOs. Samples were then harvested for RNA to measure *ENST8800* and *ZFAS1*, as well as protein for western blotting analysis. Top, schema of the ASOs used. Middle, RT-qPCR of 3 experiments. ** $p<0.01$, *** $p<0.001$. Bottom, western blot representative of 2 experiments. Signals were normalized to Tubulin beta (TUBB) and are expressed relative to the CTLASO values.

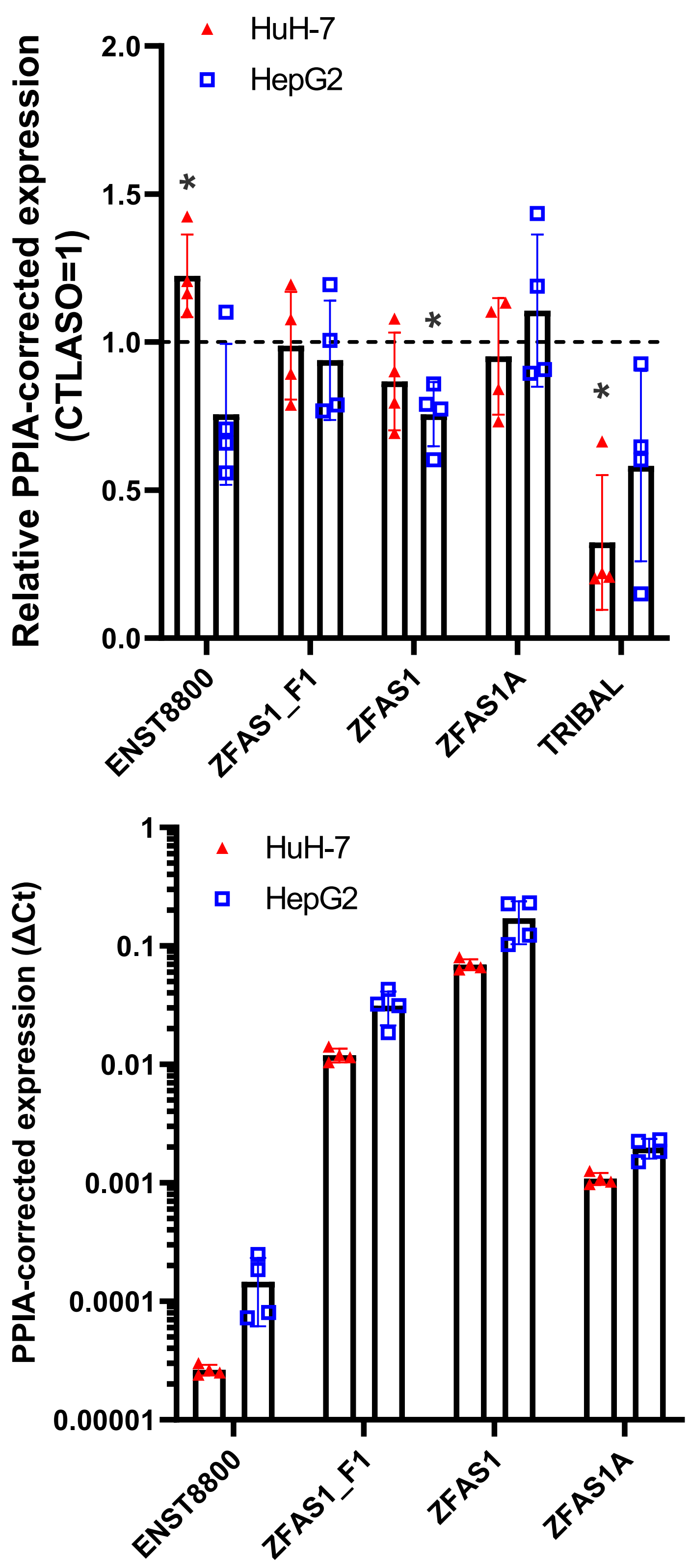

Figure S7. Minimal impact of *TRIBAL* suppression on *ZFAS1* expression in HuH-7 and HepG2. HepG2 and HuH-7 samples(n=4) treated with *TRIBAL* ASO2 or a non-target control ASO for 72 h were tested for *ZFAS1* expression by qRT-PCR. Top, relative expression of ASO2- vs CTLASO-treated samples. *PPIA* values Statistical significance shown is from a Student's test vs a hypothetical value of 1 (null hypothesis: no difference with control). * p<0.05. Bottom, *PPIA*-corrected abundance of *ZFAS1* using CTLASO values.

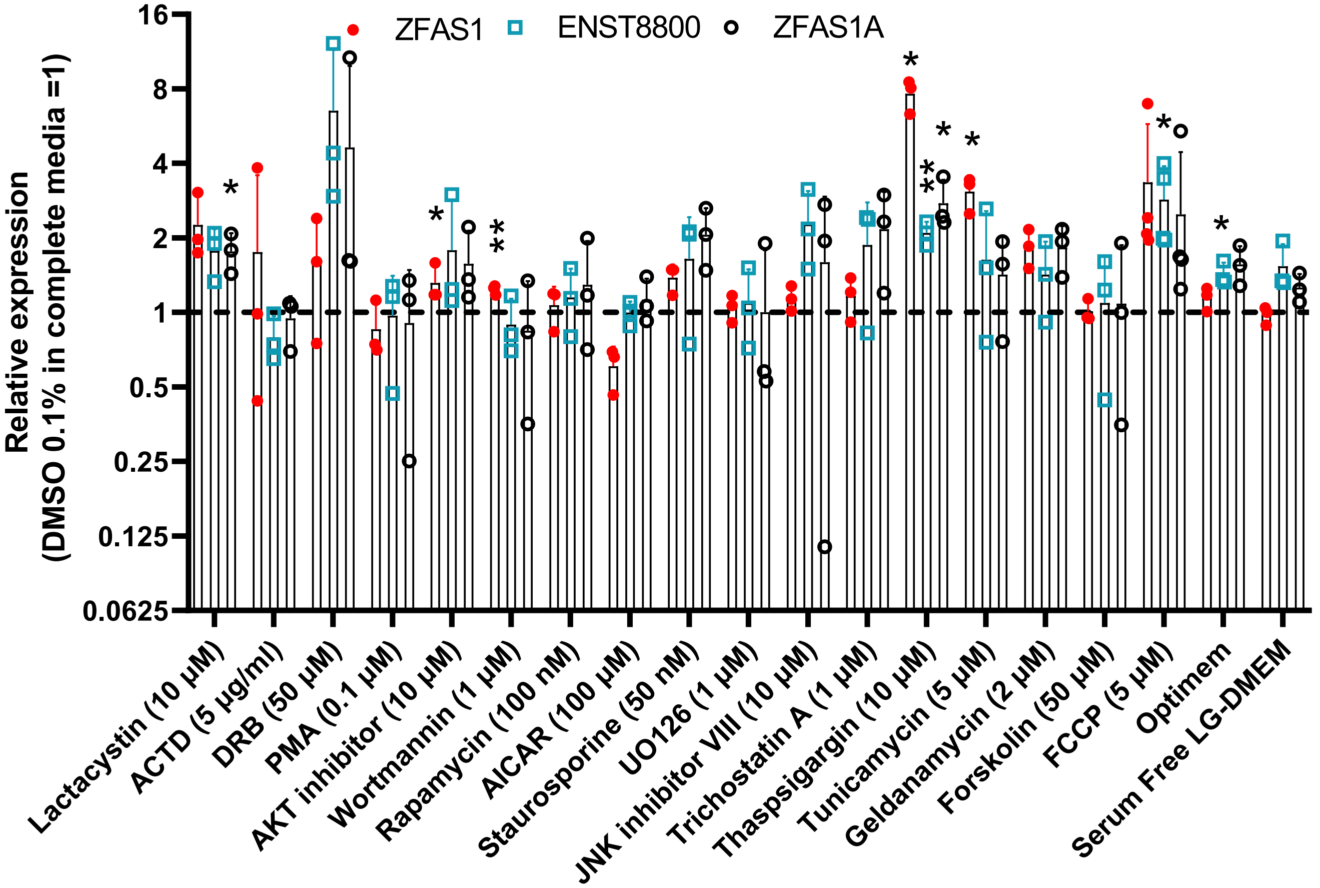

Treatment/Drug	Target	Treatment/Drug	Target
Lactacystin	Proteasome inhibitor	JNK inhibitor VIII	JNK inhibitor
ACTD	Transcription inhibitor	Trichostatin A	HDAC inhibitor
DRB	CK2 and transcription inhibitor	Thaspsigargin	ER Ca pump inhibitor
PMA	PKC activator	Tunicamycin	N-glycosylation inhibitor
AKT inhibitor (GSK690693)	AKT inhibitor	Geldanamycin	Chaperone inhibitor
Wortmannin	PI3K inhibitor	Forskolin	adenylcyclase inhibitor
Rapamycin	MTOR inhibitor	FCCP	Mitochondrial uncoupler
AICAR	AMPK activator	Optimem	Serum free media with supplements
Staurosporine	Protein Kinase inhibitor	Serum Free LG-DMEM	Serum free media
UO126	MEK 1/2 inhibitor		

Figure S8. *ZFAS1* abundance in response to a panel of modulators. RNA quantification of HepG2 cells (n=3) that were treated for 18 h in the presence of the indicated drugs and/or media. The table summarizes the canonical targets/functions impacted. * $p<0.05$, ** $p<0.01$.

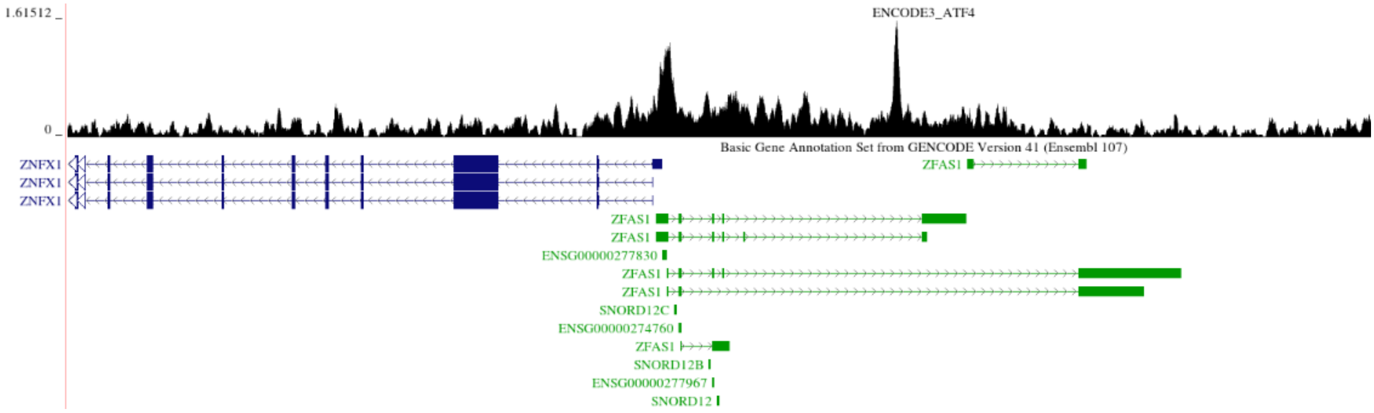

Figure S9. ATF4 is present on the *ZFAS1* promoter region and proximal to *ENST8800*. Data from the Cistrome Data Browser (http://cistrome.org/db/#/), of ENCODE3 ATF4 (PMID: 29126249) HepG2 data visualized with the UCSC browser (https://genome.ucsc.edu/cgi-bin/hgTracks).

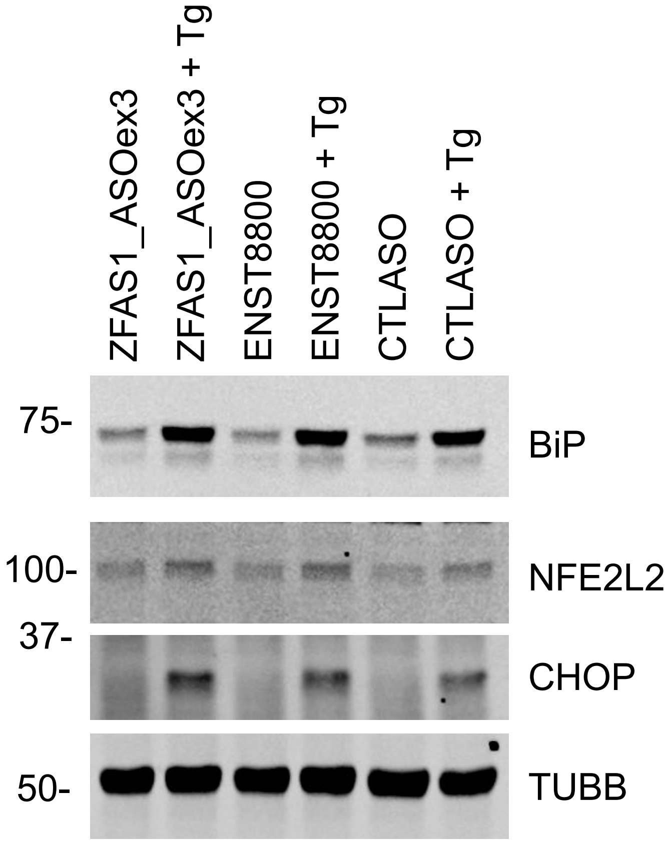

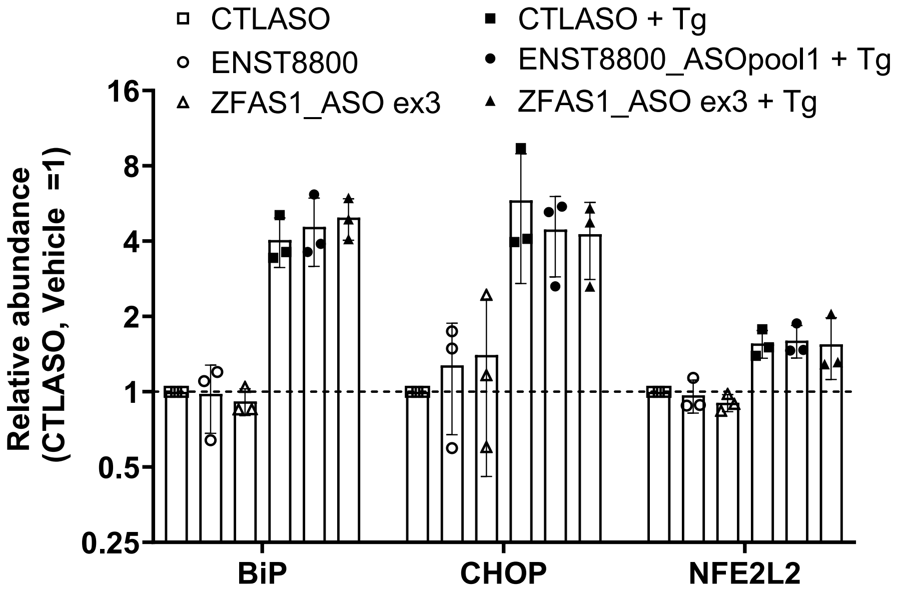

Figure S10. No significant impact of ZFAS1 suppression on the UPR effectors. Western blot analysis of ZFAS1- and ENST8800-suppressed cells, basally and in response to Tg (1 µM, 6 h). A blot representative of 3 experiments is shown on top. Quantification of three biological replicates is shown underneath, expressed relative to Tubulin beta abundance, and normalized to the CTLASO + vehicle (CTLASO) value. There was no statistically significant difference between the CTLASO and the matching ASO values (Student's t-test).

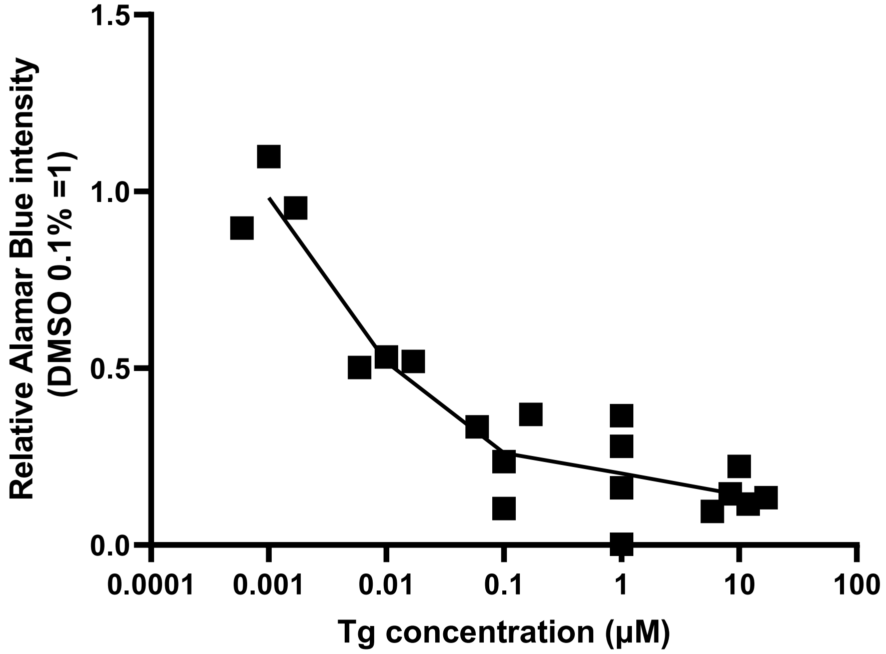

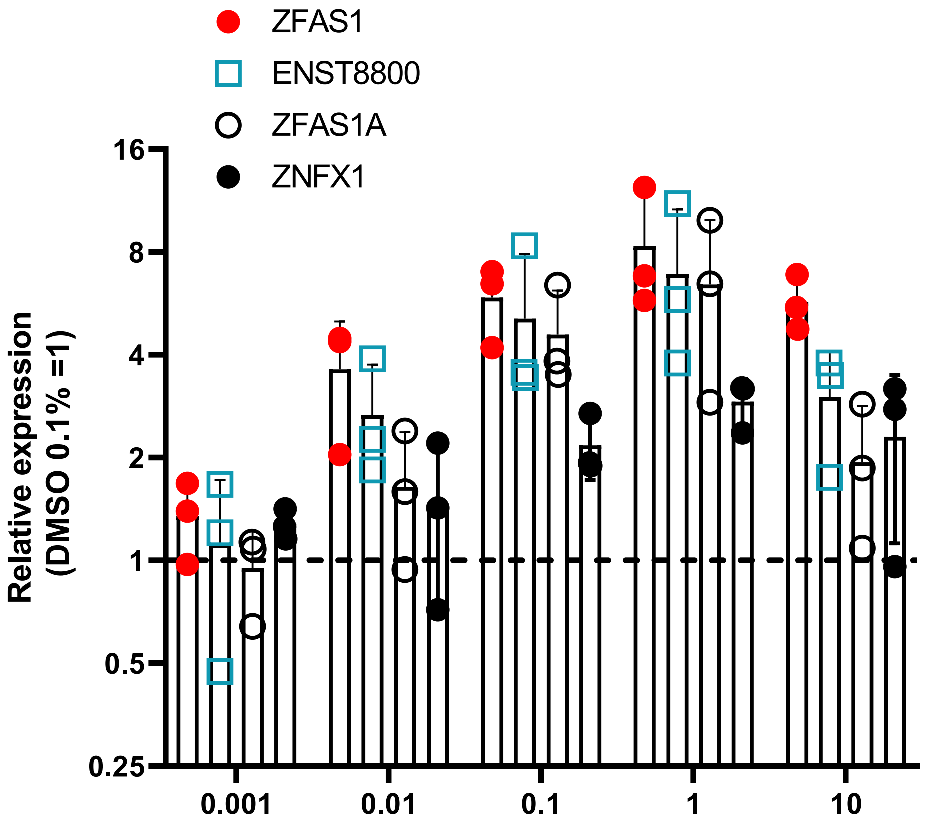

Figure S11. Thapsigargin is toxic and increases *ZFAS1* durably. HepG2 cells (n=3 or 4) were treated with the indicated doses of Tg for 48 h, and were assayed for viability measured using the Alamar assay (Top) and transcript abundance (bottom).
