# Supplementary material for: Differential regulation of ZFAS1 splice variants by endoplasmic reticulum stress in hepatocyte cell lines
Source: FEBS Open Bio. 2026 Feb 6;16(6):1115–31. doi: 10.1002/2211-5463.70185 (PMC13238724; doi:10.1002/2211-5463.70185)
Supplement: Supplementary file 2 — Supporting Information S1. List of nucleotides, antibodies used, and novel ENST8800 sequences. [file FEB4-16-1115-s002.docx]

PCR and qRT-PCR primers (forward, reverse)

ZFAS1 (F1,R1)

TTAGAGCAGCCAGCGGGTA

CTGGTTCAATCAAAGCCTGGTT

ZFAS1F1’

TTGGAAGAGGGAGTCACCAC

ENST8800 (F2, R2)

TTGAGATGCAACCAGAGCGAT

CAAGATGAAGGCCTCTTGCC

SNORD12

CCTTTGCAGCTGATGATACAGCT

GCCAATGCATCAGACAAA

SNORD12B

CTTCCTGCTGGCATATATGATGACTTAGC

GCTCAAGCTGGCATATC

SNORD12C

TGTAAATGATGACTTCACTTTT

TGTATCAGAACTAACTGGCA

PPIA

ACCGTGTTCTTCGACATTGC

TTCTGTGAAAGCAGGAACCC

TRIBAL

ACCTCCCCATGATCCAA

CATTCTGGAAGCTCTGTTGAC

ATF4

TCCAACAACAGCAAGGAGGATG

TCCAACGTGGTCAGAAGGTCATC

BiP

ACCACCTACTCCTGCGTC

TTGGAGGTGAGCTGGTTCT

CHOP

CCTGGAAATGAAGAGGAAGAATC

ACTGGAATCTGGAGAGTGAGG

NRF2

CTCATCATGATGGACTTGGAGCTG

GTCTCTTCATCTAGTTGTAACTGAG

SRP14

ACTTCCGGCTCTCACTGCTA

TCAAAGCCCTCCACAGTACC

RNU1-1

ATACTTACCTGGCAGGGGAG

CAGGGGGAAAGCGCGAACGCA

MALAT1

CCAACAGCACAGCGGTACAC

AACAGGGAAGAGAGAGGGTG

ATP5AF1

CTGGCATTAAGGCTGTGGAT

ACTGGGCAACAGTGGATCTC

Antisense oligonucleotides

ASOs were modified (nucleotide sequence 5’ to 3’) 2’-O-(2-methoxy) ethyl-modified gapmers (10 nucleotides; 5 on either side) with a phosphorothioate backbone. Obtained from Integrated DNA Technologies (IDT). Top, actual sequence; bottom, oligonucleotide as ordered.

CTLASO (non-target control):

CCTTCCCTGAAGGTTCCTCC

/52MOErC/*/i2MOErC/*/i2MOErT/*/i2MOErT/*/i2MOErC/*C*C*T*G*A*A*G*G*T*T*/i2MOErC/*/i2MOErC/*/i2MOErT/*/i2MOErC/*/32MOErC/

TRIBAL ASO2:

GGTGTGCAGGGTACCCTCTC

/52MOErG/*/i2MOErG/*/i2MOErT/*/i2MOErG/*/i2MOErT/*G*C*A*G*G*G*T*A*C*C*/i2MOErC/*/i2MOErT/*/i2MOErC/*/i2MOErT/*/32MOErC/

TRIBAL ASO9:

AGGCCGTGGTCTCAGAAGCA

/52MOErA/*/i2MOErG/*/i2MOErG/*/i2MOErC/*/i2MOErC/*G*T*G*G*T*C*T*C*A*G*/i2MOErA/*/i2MOErA/*/i2MOErG/*/i2MOErC/*/32MOErA/

ZFAS1EXON2_1 (ASO9):

GTTGTAGATGTCTGCACGTG

/52MOErG/*/i2MOErT/*/i2MOErT/*/i2MOErG/*/i2MOErT/*A*G*A*T*G*T*C*T*G*C*/i2MOErA/*/i2MOErC/*/i2MOErG/*/i2MOErT/*/32MOEr/G

ZFAS1EXON2_2 (ZFAS ASO10):

TCCATTCTGTACCCGCTGGC

/52MOErT/*/i2MOErC/*/i2MOErC/*/i2MOErA/*/i2MOErT/*T*C*T*G*T*A*C*C*C*G*/i2MOErC/*/i2MOErT/*/i2MOErG/*/i2MOErG/*/32MOEr/C

ZFAS1EXON3_1 (ZFAS ASO7):

GGTTCAATCAAAGCCTGGTT

/52MOErG/*/i2MOErG/*/i2MOErT/*/i2MOErT/*/i2MOErC/*A*A*T*C*A*A*A*G*C*C*/i2MOErT/*/i2MOErG/*/i2MOErG/*/i2MOErT/*/32MOErT/

ZFAS1EXON3_2 (ZFAS ASO8):

CTTCCAACACCCGCATTCAT

/52MOErC/*/i2MOErT/*/i2MOErT/*/i2MOErC/*/i2MOErC/*A*A*C*A*C*C*C*G*C*A*/i2MOErT/*/i2MOErT/*/i2MOErC/*/i2MOErA/*/32MOErT/

ENST8800pool1 (ZFASASO1 + ZFASASO3 + ZFASASO5)

ZFASASO1

ACCTATCCCTAGGTGCACAT

/52MOErA/*/i2MOErC/*/i2MOErC/*/i2MOErT/*/i2MOErA/*T*C*C*C*T*A*G*G*T*G*/i2MOErC/*/i2MOErA/*/i2MOErC/*/i2MOErA/*/32MOErT/

ZFASASO3

GTTGCCAATACCTGGGAAGG

/52MOErG/*/i2MOErT/*/i2MOErT/*/i2MOErG/*/i2MOErC/*C*A*A*T*A*C*C*T*G*G*/i2MOErG/*/i2MOErA/*/i2MOErA/*/i2MOErG/*/32MOErG

ZFASASO5

GACGGACTTGTACTTCCCTC

/52MOErG/*/i2MOErA/*/i2MOErC/*/i2MOErG/*/i2MOErG/*A*C*T*T*G*T*A*C*T*T*/i2MOErC/*/i2MOErC/*/i2MOErC/*/i2MOErT/*/32MOErC

ENST8800pool2 (ZFASASO2 + ZFASASO4 + ZFASASO6)

ZFASASO2

CCCTGTGGTCAAGCATTTAG

/52MOErC/*/i2MOErC/*/i2MOErC/*/i2MOErT/*/i2MOErG/*T*G*G*T*C*A*A*G*C*A*/i2MOErT/*/i2MOErT/*/i2MOErT/*/i2MOErA/*/32MOErG/

ZFASASO4

GATCGTAGGGAGCAAAGGCT

/52MOErG/*/i2MOErA/*/i2MOErT/*/i2MOErC/*/i2MOErG/*T*A*G*G*G*A*G*C*A*A*/i2MOErA/*/i2MOErG/*/i2MOErG/*/i2MOErC/*/32MOErT

ZFASASO6

AAGTGACAATGTCAGTAGCC

/52MOErA/*/i2MOErA/*/i2MOErG/*/i2MOErT/*/i2MOErG/*A*C*A*A*T*G*T*C*A*G*/i2MOErT/*/i2MOErA/*/i2MOErG/*/i2MOErC/*/32MOErC

Antibodies

MLXIPL (ChREBP): Rabbit pAb Antibody #58069 (Cell Signaling Technology)

HNF4A: Rabbit mAb #3113 (C11F12; Cell Signaling Technology)

TUBB: Mouse mAb GTX11307 (D66; GeneTex)

CHOP: Mouse mAb #2895 (L63F7; Cell Signaling Technology)

BiP: Rabbit mAb #3177 (C50B12; Cell Signaling Technology)

NFE2L2/NRF2: Rabbit mAb #20733 (E5F1A; Cell Signaling Technology)

ENST8800 sequences

New exons and longer exon 1 sequences identified:

>Exon 2

*CCAGCTGATGCTTAGGAAAATAGAAAAGAACCTACGTGATTATCAGGGCAGGTCCCCCGATAAGTAATAGTCATCTTCATCCGAGTGAAAAGGAATAAACTAGTGATTGTGCAACAACTTGGATGAACCCCAGAGATGCGATGCTGACTGAGGGAAGCCAGCCTCAAATGGTTACATGCTGTGTGATTCCATTTATTTGACATTTTGGAAAAGACAA*

>Exon 3a

*AGGCTGGAGTGCAATGGTGCGATCTTAGCTCACTGCAGCCTCCACCTTCCGGGTTCAAGCAGTCCTCCCGCCTCAGCCTCCTGAGTAGCTGTGACTGCAG*

>Exon 3b

*AGGGTCTTGCTCTGTCGCCCAGGCTGGAGTGCAATGGTGCGATCTTAGCTCACTGCAGCCTCCACCTTCCGGGTTCAGGCAATCCTCCCGCCTCAGCCTCCTGAGTGGCTGTGACTGCAG*

>Longer exon 1 clone #1

TTTTCASACTTATTTGTGCATTTGACTTAACATGTCCAAAACTAAATGCCTGATGCTTCACTCACCTAACCCCATTCCCTGCAAGTCTGTGCTTCCTGCGATCTTC

>Longer exon 1 clone #2

GCCAGCGTAGCAATCAACTGAGGTTGAACCATTATTGTGGGAGGAGTTACCCAACAACCATGTGAGTTTGTAAGCACGTCTTTCTGTAGCGGAGGCTTGATGACTACCACCCCAGCAGACCTCTCGGAGATGCTTG

>Longer exon 1 clone #3

TGGGAGGAGTTACCCAACAACCATGTGAGTTTGTAAGCACGTCTTTCTGTAGCGGAGGCTTGATGACTACCACCCCAGCAGACCTCTCGGAGATGCTTG
