# Supplementary material for: Differential regulation of ZFAS1 splice variants by endoplasmic reticulum stress in hepatocyte cell lines
Source: FEBS Open Bio. 2026 Feb 6;16(6):1115–31. doi: 10.1002/2211-5463.70185 (PMC13238724; doi:10.1002/2211-5463.70185)

Sequential probing (TUBB + HNF4A, then MLIXPL; same order as Fig S6)

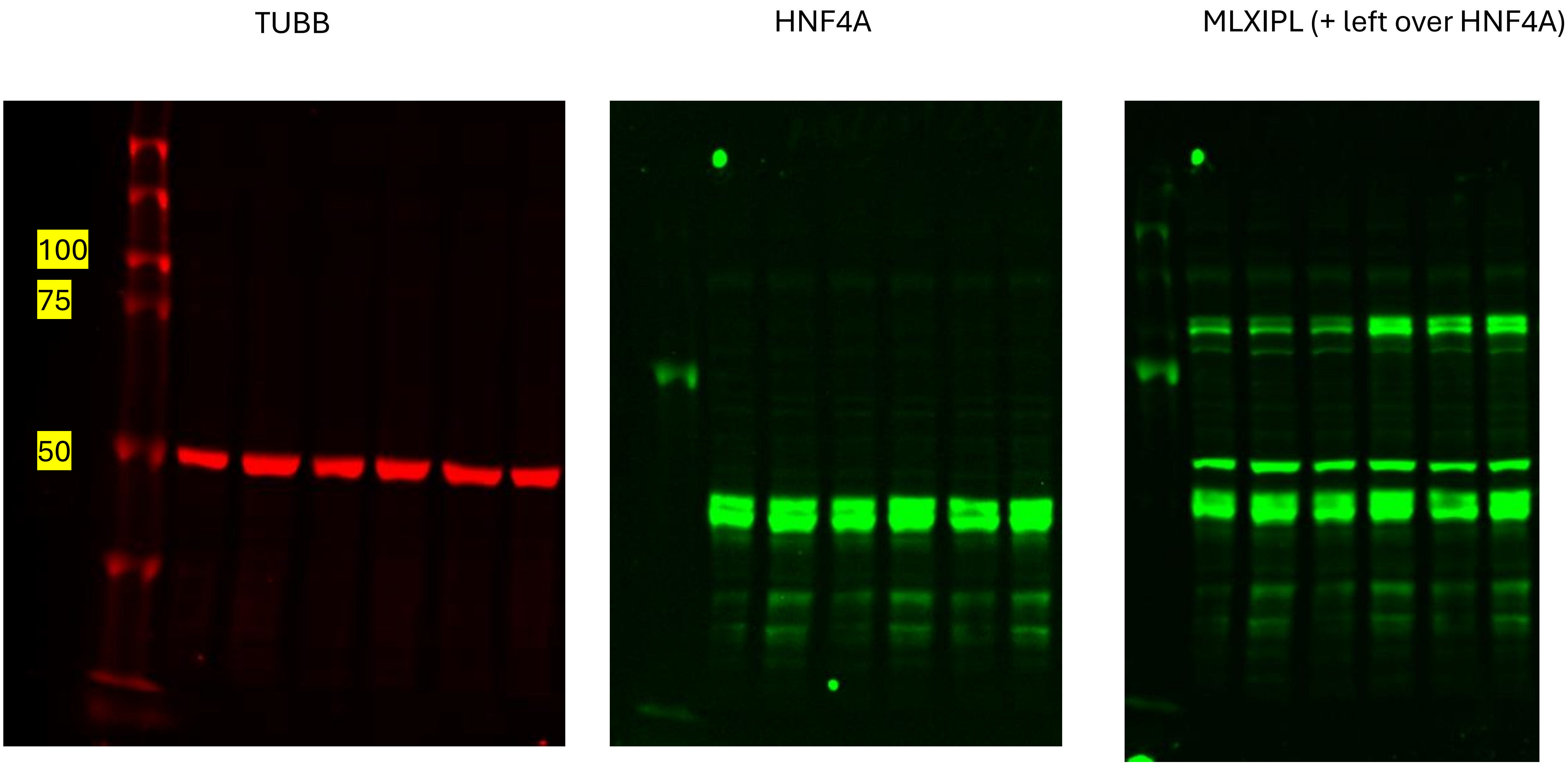

Fig S10 original blots

Sequential probing (BiP, then TUBB, then NFE2L2 + CHOP)

BiP

TUBB

NFE2L2 (green) + CHOP (red)

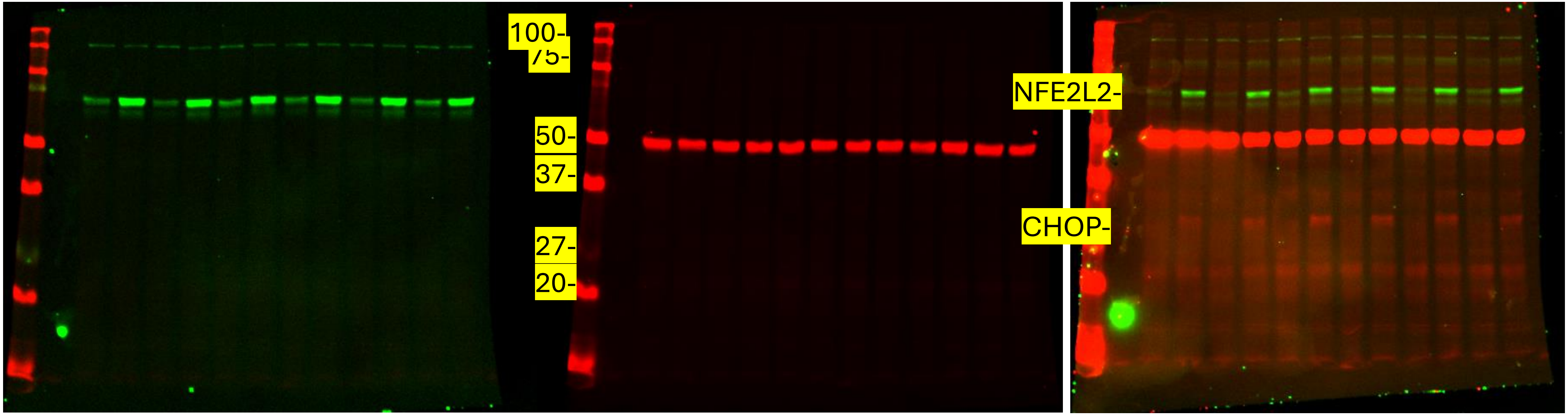

Supplement: Supplementary file 3 — Data S1. Uncropped blots. Complete versions of the blots shown in the manuscript. [file FEB4-16-1115-s001.pdf]
